# Connectivity and Age of Restored Atlantic Forest Fragments Drives Composition and Functionality of the Fungal Community in the Leaf Litter Layer

**DOI:** 10.1111/mec.70325

**Published:** 2026-03-24

**Authors:** Guilherme Lucio Martins, Dina in 't Zandt, Luis Fernando Merloti, Wanderlei Bieluczyk, Gabriel Silvestre Rocha, Robert Timmers, Ricardo Ribeiro Rodrigues, Siu Mui Tsai, Wim H. van der Putten

**Affiliations:** ^1^ Center for Nuclear Energy in Agriculture (CENA) University of São Paulo (USP) Piracicaba São Paulo Brazil; ^2^ Luiz de Queiroz College of Agriculture (ESALQ), University of São Paulo (USP) Piracicaba São Paulo Brazil; ^3^ Department of Terrestrial Ecology Netherlands Institute of Ecology (NIOO‐KNAW) Wageningen the Netherlands; ^4^ Department of Ecology, Radboud Institute for Biological and Environmental Sciences (RIBES) Radboud University Nijmegen the Netherlands; ^5^ Department of Microbial Ecology Netherlands Institute of Ecology (NIOO‐KNAW) Wageningen the Netherlands; ^6^ Ecology & Biodiversity Group, Department of Biology Utrecht University Utrecht the Netherlands; ^7^ Department of Nematology Wageningen University & Research Wageningen the Netherlands

**Keywords:** ecosystem services, forest chronosequence, fungal guilds, fungi sequencing, landscape connectivity, litter saprophytes

## Abstract

The restoration of biodiversity and functional tropical forests is critical to mitigating global biodiversity losses. Aboveground, increasing the connectivity of regenerating forest fragments facilitates the recolonization of tropical forest biodiversity. However, restoring functional ecosystems also requires the recovery of decomposition processes as these are essential in shaping aboveground biodiversity. Therefore, we investigate the role of forest connectivity in restoring the composition and functioning of fungal communities in the leaf litter layer during a chronosequence of forest restoration. In the Brazilian Atlantic Forest, we studied secondary forests regrown between 18 and 55 years after deforestation and different levels of forest connectivity and compared their litter to recently abandoned pastures and undisturbed primary forests. We quantified how forest age and connectivity between fragments influenced the litter fungi composition in relation to tree diversity, litter chemistry and litter isotopes. We show that fungal composition was highly heterogeneous in forest litter, whereas pasture litter exhibited a more homogeneous community. Moreover, forest connectivity had stronger effects on litter fungal composition compared to forest age. Connectivity promoted wood saprotrophs and endophytes, while suppressing soil saprotrophs, with its effects being more evident during later stages of restoration. Fungal guilds such as endophytes and saprophytes were primarily influenced by tree diversity and leaf litter chemistry. We conclude that forest connectivity promotes the re‐establishment of saprophytic fungi capable of decomposing recalcitrant litter substrates, driven mainly by enhancing tree diversity and litter quality. Practical implications of increasing connectivity may relate to forest resilience in front of future climate change scenarios.

## 1 Introduction

The Brazilian Atlantic Forest biome encompasses 1.6 million hectares and stands as one of the global biodiversity hotspots, harbouring diverse and endemic flora and fauna and storing an estimated 315–460 Mg of carbon per hectare in above‐ and belowground biomass (Vieira et al. 2011). However, centuries of deforestation driven by urbanization, agriculture and livestock expansion have fragmented native habitats, reducing connectivity and isolating populations within the remaining forest fragments. This fragmentation limits the movement of fauna and flora, increases the risk of inbreeding and local extinction, and ultimately threatens the functional biodiversity and ecosystem stability (Saura et al. 2017). The current primary forest is fragmented and covers only 11%–16% of its original area, and in many cases, it is vastly degraded (Rezende et al. 2018; de Lima et al. 2020). Since 1970, efforts to restore Atlantic Forest (Rodrigues et al. 2009) aimed to preserve the biodiversity of primary forests while reconnecting the remaining fragments to support species dispersal, forest regeneration, ecosystem resilience and climate change mitigation (Poorter et al. 2021; Rother et al. 2023). These efforts have mainly focused on converting degraded pasture into secondary forests, as pastures are the main cause of connectivity loss. In this context, connectivity metrics, such as surrounding land use and the percentage of forest cover, provide valuable tools for assessing the regeneration potential of forest fragments (César et al. 2021).

While current restoration efforts focus primarily on restoring aboveground biodiversity, studies on belowground diversity have increased over the last few years. For instance, belowground microbes and soil fauna are important drivers of organic matter decomposition and recovery of soil biogeochemical processes (Wang and Kuzyakov 2024; Vivian et al. 2025; Rivera et al. 2025), thereby supporting forest restoration. Yet, these processes have not been examined in the context of different levels of forest connectivity during restoration. Forest connectivity can increase soil biodiversity by direct and indirect effects, with direct effects coming from ecological corridors that enhance the dispersal of aboveground species, increasing the diversity of tree species in forest fragments (Salviano et al. 2021), as well as increasing propagules (i.e., leaves, fruits and seeds) by wind and animal, resulting in increasing the fragment size and connectivity (Timmers et al. 2025; Hatfield et al. 2024). In contrast, indirect effects of forest connectivity on microbial biodiversity include the greater plant diversity that in turn increases litter carbon accumulation and provides higher‐quality litter substrate to promote microbial activity for organic matter cycling (Zhang et al. 2022).

As a result, it is expected that increasing forest connectivity may influence litter chemistry and fungal composition, thereby affecting the functions performed by the fungi community in the litter layer. For example, litter decomposition by saprotrophic bacteria and fungi transfer carbon and mineral nutrients from plant litter to the soil foodweb (Baldrian 2017; Liu et al. 2023). Such nutrients become available for plant uptake, facilitating tree growth and triggering a cascade of symbiotic interactions that revitalize forest biodiversity, which may eventually reach levels comparable to those of primary forests in the long‐term (Bardgett and Van Der Putten 2014; Chai et al. 2019). However, there is a major knowledge gap on the relationship between connectivity of forest fragments, leaf litter attributes such as biomass and nutrient concentration, and the composition of fungal groups in the leaf litter of these fragments. Understanding how forest connectivity influences the composition and functionality of leaf litter fungi may help predict and possibly steer the recovery of forests in restoration programs.

In the present study, we examined the composition and putative functioning of fungi present in Brazil's Atlantic Forest litter layer in relation to time since the start of forest restoration, and connectivity with nearby forest fragments. Pastures were considered as a restoration starting point (0 years) and Primary forests were considered the endpoint (100 years) of forest restoration. We quantified how forest age and connectivity influence litter chemistry, litter biomass and fungal composition. The research was carried out in a chronosequence of restored forests with varying levels of connectivity among forest fragments. The litter was characterized by means of chemical, molecular, and isotopic analyses, while forest connectivity was measured using satellite imagery. We tested the hypotheses that: (i) leaf litter chemistry and its fungal composition are different for each land‐use type; (ii) older and more connected forests present higher leaf litter biomass and higher fungal β‐diversity than younger and less connected ones; (iii) tree diversity is the most important pathway that mediates the abundance of fungal guilds due to its litter composition.

## 2 Material and Methods

### 2.1 Study Sites and Experimental Design

The study was conducted in the mesoregion of Piracicaba, São Paulo, Brazil (Table S1). The region's climate is humid subtropical (Cfa, Köppen) with a semi‐deciduous seasonal tropical forest vegetation characterized by hot and humid summers and dry winters, with an average annual precipitation of 1273 mm (Alvares et al. 2013). In this region, 15 sites were selected to represent a spatial–temporal gradient of forest restoration including variation in both forest age and connectivity (Table 1). We used three pasture areas adjacent to secondary forests, managed like before the forest was restored, to represent the starting point of forest restoration. In contrast, three primary forests, each at least 100 years old, represented the target of restoration. There were nine secondary forests, categorized into early (18–23 years), intermediate (31–38 years), and late (43–55 years) stages of forest restoration. All secondary forests were previously abandoned pastures and were restored by passive restoration (Brancalion et al. 2016), which consists of allowing natural regrowth without planting seedlings or fertilization, only preventing disturbances from humans and cattle grazing. The forest fragments varied in proximity to other forest patches, representing different levels of connectivity. The geographic coordinates of all sites are provided in the (Table S1).

**TABLE 1:** Description of the sites used in this study at the sampling time.

Land‐use	Age (years)	Age class	Forest connectivity (%)^a^	Connectivity class
Primary forest	> 100	Old‐growth	46	High
			32	Mid
			13	Low
Secondary forest	43–55	Late	32	High
			29	Mid
			13	Low
	31–38	Intermediate	36	High
			18	Mid
			1.7	Low
	18–23	Early	38	High
			31	Mid
			13	Low
Pasture			0	No connectivity
	0	—	0	No connectivity
			0	No connectivity

^a^ Forest connectivity was calculated as the percentage of native forest cover (native or planted) within a 1 km radius around the plot using satellite images.

Forest age was provided by researchers who were in charge of the restoration project and confirmed by the stakeholders responsible for each area under restoration. This information was further validated using a chronology of satellite images (MapBiomas—(https://mapbiomas.org/)) and historical land cover maps from 1962 to 2008 (César et al. 2021). Forests undergoing restoration were classified into three maturity classes (early/intermediate/late) based on visual indicators such as forest formation type, canopy cover, the presence of late successional tree species, and species with distinct and complementary ecological roles (Rodrigues et al. 2011; Carlucci et al. 2020; Rother et al. 2023).

A proxy of forest connectivity was calculated using high‐resolution (5 m) satellite images and MapBiomas forest cover data. Connectivity was defined as the proportion of forest cover within a 1 km radius around each sampling plot, serving as an indicator of habitat availability in the landscape (Rezende et al. 2018; Timmers et al. 2025). Monoculture plantations, such as *Eucalyptus*, were excluded from the calculation, as they do not represent real forest ecosystems. This landscape‐scale connectivity metric offers a more robust estimate of ecological connectivity than patch‐level metrics such as fragment size or isolation as it combines both metrics into a single one (Fahrig 2013). Connectivity was categorized into three levels: low (1.7%–13% forest cover), mid (18%–32%), and high (33%–46%). Each age class included one site of each connectivity level (Table 1).

### 2.2 Forest Inventory and Litter Sampling

Forest inventory was conducted by establishing a 30 × 30 m plot in each site, located at least 50 m away from forest edges and excluding glades and areas impacted by human trails. All trees with a diameter at breast height (DBH) greater than 5 cm were counted and identified to species level (see Supporting Information S1). Plant taxonomic diversity was assessed by the Shannon‐Wiener index using the *vegan* package (Oksanen et al. 2019) with the R language. A complete list of tree species and their abundances is provided in Table S5.

Litter sampling was conducted in May 2022, at the end of the wet season. For DNA extraction, a mixture of decaying leaf litter was collected from the forest floor, carefully avoiding soil, branches, and wood pieces. Leaf litter from different tree species was not distinguished during sampling. Five samples were collected from each plot (30 × 30 m), one from each corner and one from the center, using 50 mL sterile plastic tubes, each containing approximately 5 g of leaf litter. Samples were then stored and transported in iceboxes to the laboratory for storage in a freezer at −20°C.

For the litter dry biomass weight and chemical analysis, square plastic frames of 25 × 25 cm were used to collect leaf litter material (five replicates per plot). All collected material was carefully taken to exclude soil, branches, and wood pieces. Litter was stored in paper bags and dried at 50°C for 48 h or until constant weight was achieved.

### 2.3 Litter Chemical Analysis

Dry leaf litter samples were ground using a knife mill to achieve a particle size lower than 0.25 mm, ensuring sample homogeneity for element content analysis. Five samples were analysed per plot. The ground litter was analysed for total macro (P, K, Ca, Mg, and S) and micronutrient (Fe, Cu, Zn, and Mn) content, according to Malavolta et al. (1989). Briefly, the element concentrations were determined from the extract obtained by digesting the litter material in a mixture of HNO_3_ and HClO_4_. P and S were quantified by colorimetry, while other elements were quantified by atomic absorption spectroscopy (PerkinElmer 3100, USA).

Litter C and N concentration and ^15^N and ^13^C isotopes were analysed using an elemental analyser (Carlo Erba, CHN1110; Milan, Italy) coupled to a mass spectrometer (Thermo Fisher, Delta Plus; Bremen, Germany). Stable isotope results were expressed as δ^13^C and δ^15^N (‰) using international standards (Vienna PeeDee Belemnite—V‐PDB for C [NBS19 and NBS22] as a reference for ^13^C and composition of atmosphere for N^2^ [IAEA‐N1 and IAEA‐N2] as a reference for δ^15^N (Farquhar et al. 1982)). The delta (δ) values were calculated using the following equation: *δX* = [(R_sample_/R_standard_) −1] multiplied by 1000, where *X* refers to ^13^C or ^15^N, and R_sample_ and R_standard_ are the ^13^C/^12^C or ^15^N/^14^N, ratios of sample and standard, respectively.

### 2.4 Litter DNA Extraction, Sequencing of ITS Region and Bioinformatics

DNA was extracted from litter samples using the protocol by England et al. (2004). Briefly, 1.0 g of frozen litter was incubated in 10 mL of sterile 0.5% (w/v) phosphate buffer solution (PBS) at pH 8.0 and shaken for 16 h at 250 rpm and then centrifuged at 13,000 × *g* for 20 min at 4°C. The resulting pellet underwent DNA extraction using the DNeasy PowerLyzer PowerSoil Kit (Qiagen, Hilden, Germany) according to the manufacturer's instructions. DNA integrity was assessed through 1% (w/v) agarose gel electrophoresis, and DNA quantity was measured using a NanoDrop 2000c (Thermo Fisher, Waltham, USA). When necessary to remove contamination from PCR inhibitors, samples were purified using the Illustra GFX Purification Kit (GE Healthcare, Buckinghamshire, UK) according to the manufacturer's protocol.

A total of 75 samples were sequenced using the fungal internal transcribed spacer (ITS) region with the primers ITS3‐2024F/ITS4‐2409R (White et al. 1990). The library was constructed using the Illumina NextSeq 1000/2000 P1 Reagent kit (Illumina, San Diego, USA) using 35 cycles, and amplicon fragment size (~300 bp) was confirmed by gel electrophoresis. PCR reactions followed the Nextera XT Kit protocol (Illumina, San Diego, USA), and DNA amplification was confirmed by gel electrophoresis. Purified amplicons were equimolarly pooled, and the library's final concentration was determined using a SYBR green quantitative PCR assay with primers specific to the Illumina adapters Kapa (KAPA Biosystems, Boston, USA).

ITS gene sequencing analysis was performed using the pipeline from *DADA2* (Callahan et al. 2016) package. Briefly, the primers from the demultiplexed data were removed, and data were trimmed and filtered to remove low‐quality sequences, and the denoising inference step was performed (based on the learn error step). The forward and reverse sequences were merged (resulting in 2 × 250 bp paired‐ended reads), and the chimeric sequences were removed based on the ‘consensus’ method. Amplicon Sequencing Variants (ASVs) were assembled from the retained sequences, and taxonomic assignments were made according to the UNITE database (v. 8) (Nilsson et al. 2019). Data was rarefied to the lowest sample size only for diversity analysis, to reduce overestimation due to differences in sample sizes. The sequences are available at the NCBI Sequence Read Archive under the identification PRJNA1296929.

### 2.5 Statistical Analysis

All statistical analyses were conducted in R (version 4.5.1). To test the first hypothesis, we performed a Principal Component Analysis (PCA) using the *factoextra* package to reduce the dimensionality of litter chemical attributes and compare differences between land‐use types. Variables included macronutrients (N, P, K, Ca, Mg and S), micronutrients (Cu, Zn and B) and isotopic signature of the litter (*δ* ^13^C and *δ* ^15^N). Fungal composition was transformed into centered log‐ratio (*clr*), and the β‐diversity was calculated using non‐metric multidimensional scaling (NMDS) using the *vegan* package. Differences among land‐use types were compared using Permutational Multivariate Analysis of Variance (PERMANOVA) via the *pairwiseAdonis* function. We used Random Forest analysis with the *microeco* package (Liu et al. 2021) to identify fungal biomarker groups within each land‐use type. Fungal functional groups were assigned to ecological guilds using the FUNGuild database (Nguyen et al. 2016), considering only classifications marked as ‘probable’ and ‘highly probable’. Although functional annotation of tropical fungi remains incomplete in global databases, 65.2% of the fungal community was successfully assigned to guilds, providing sufficient coverage for downstream analyses.

To test our second hypothesis, we used linear mixed‐effects models (LMMs) with the *lme4* package (Bates et al. 2015) to investigate the effects of forest age and forest connectivity on litter chemistry and biomass, and fungal composition (β‐diversity) along the chronosequence of forest restoration. Forest age and forest connectivity were included as interacting effects as predictors, and litter parameters and fungal community were used as response variables and were tested individually, and plot was included as a random factor. Model assumptions, including residual normality, errors homoscedasticity, collinearity and absence of influential points and outliers, were checked using the *performance* package (Lüdecke et al. 2021). If assumptions were violated, data were square‐root transformed to meet model assumptions. We further used Spearman correlation analysis with the *corrplot* package to assess relationships among fungal guilds, tree diversity and litter chemistry, setting a threshold of *p* < 0.01.

To test our third hypothesis, a structural equation model (SEM) was constructed to assess the potential pathways through which forest age and forest connectivity influence tree diversity, litter biomass, litter chemical composition and the abundance of fungal guilds. The conceptual model was developed based on the hypothesis that increasing forest age and connectivity may promote higher tree diversity, which in turn affects litter chemistry and fungal guild composition (Figure S1, Table S2). This framework allows for the evaluation of direct relationships among forest age and connectivity, tree diversity, litter attributes, and abundance of fungal guilds, with indirect relationships being mediated by other variables. Specifically, we tested whether forest connectivity could indirectly affect fungal guild abundance by affecting tree diversity or by altering litter biomass and chemical composition as a function of forest age. For that, we included restoration age and surrounding forest cover as predictors for forest age and forest connectivity, respectively. Pastures were considered a restoration starting point (0 years), and primary forests were considered the endpoint (100 years). The first PCA axis was used as a proxy of litter chemistry composition, while the first NMDS axis was used as a proxy of litter fungal composition. Fungal guilds were included based on their relative abundance among classified fungal taxa.

We expected that older and more connected forests would correlate with higher tree diversity and greater leaf litter biomass. Increased tree diversity was expected to lead to more chemically diverse litter, influencing fungal guild composition by providing more heterogeneous substrates. Furthermore, forest age and connectivity were expected to affect fungal guild abundance through both direct and indirect pathways. Given that fungal guilds are influenced by vegetation, climate, and resource availability, we assumed autocorrelations between litter biomass and litter chemistry and between the abundance of saprophytic and endophytic fungi. Therefore, these variables were thus included as correlated error in the SEM.

The SEM was tested using the *piecewiseSEM* package (Lefcheck 2016), which enables the evaluation of individual path analyses. Each path was modelled using linear mixed‐effects models, with plot included as a random effect, and the model assumptions were checked again with the *performance* package. The global model fit was assessed using the Fischer's C statistics (*p* > 0.05), Akaike Information Criterion (AIC), and the goodness‐of‐fit index (GFI). For all models, standardized path coefficients (based on variable standard deviations) were calculated to allow comparison of the relative strength of each path within the SEM.

## 3 Results

### 3.1 Fungal Biomarkers and Fungal Guilds Across Pastures and Forests

We identified a total of 2693 fungal ASVs, with an average of 87,342 reads per sample. Two phyla dominated the fungal community in the litter layer: Ascomycota, which was significantly more abundant in pasture litter compared to primary and secondary forests (Figure S2), and Basidiomycota, which was more abundant in primary forest litter, followed by secondary forest and pasture litter. Together, these two phyla represented over 90% of the total fungal community.

Using random forest models, we identified distinct fungal biomarkers associated with each litter type (Figure S3). In primary forest litter, the class Sordariomycetes (Ascomycota) was the most important and abundant biomarker. In contrast, Leotiomycetes (Ascomycota) and Rhizophlyctidomycetes (Chytridiomycota) were the most important biomarkers for secondary forest litter, despite their relatively low abundance. In pasture litter, the most important biomarkers were Ustilaginomycetes (Basidiomycota), while Dothideomycetes (Ascomycota) was the most abundant.

We also characterized the functional composition of fungal guilds across litter types (Figure S4). The most dominant guilds were, respectively, plant pathogens, wood saprotrophs, endophytes and fungal parasites, which together comprised over 80% of the classified fungal guilds. Plant pathogens were more prevalent in primary forest litter, followed by secondary forest and pasture litter. Wood saprotrophs and endophytes were also more abundant in primary and secondary forest litter than in pasture litter. In contrast, pasture litter presented a higher abundance of soil saprotrophs, plant saprotrophs and litter saprotrophs compared to primary and secondary forest litter.

### 3.2 Litter Chemistry and Fungal Composition Are Affected by Land‐Use

**FIGURE 1:**
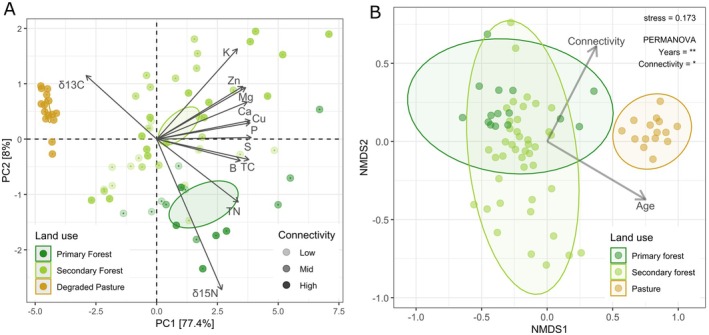
(A) The chemical composition of the litter layer under different land‐use types represented by a Principal Component Analysis (PCA). Ellipses show the confidence interval (*p* < 0.05). (B) Litter fungal composition under different land‐use types represented by a non‐metric multidimensional scaling (NMDS). Arrows indicate the effects of connectivity and age on fungal β‐diversity. The ellipses represent the confidence intervals for land‐use types (*p* < 0.05).

We analysed litter chemical attributes across different land‐use types by PCA and showed that the first two PCA axes accounted for 85.4% of the observed variation in litter characteristics (Figure 1A). Forests with high, mid, and low connectivity did not show a clear separation for litter chemical parameters. The first axis separated primary and secondary forest litter from pasture litter. This separation was related to litter total N, S, and P content, which were higher in primary and secondary forests than in pasture. Pasture litter was also characterized by the lowest nutrient contents and highest δ^13^C values. The second axis separated the primary forest litter from that of the secondary forest and pasture. The primary forest had the highest δ^15^N and B values, and the secondary forest litter had the highest content of Zn (Figure 1A and Figure S5).

We observed significant differences in fungal community composition across land‐uses (Figure 1B). Fungal composition of litter from both primary and secondary forests differed significantly from pastures (*p* < 0.001), whereas differences between the primary and secondary forest were smaller compared to differences from pasture litter. Forest age had a greater influence on fungal composition of litter than forest connectivity. Land‐use type (pasture vs. primary forest vs. secondary forest) explained 15.4% of the variation in fungal composition, while forest age and connectivity explained 4.4% and 3.2%, respectively. The fungal composition in secondary forests was highly heterogeneous, with little overlap in species abundance across samples. In contrast, fungal communities in pasture litter showed greater similarity, meaning they shared a more similar abundance of species across samples.

### 3.3 Both Forest Age and Connectivity Drives Litter Attributes and Fungal Guilds

**FIGURE 2:**
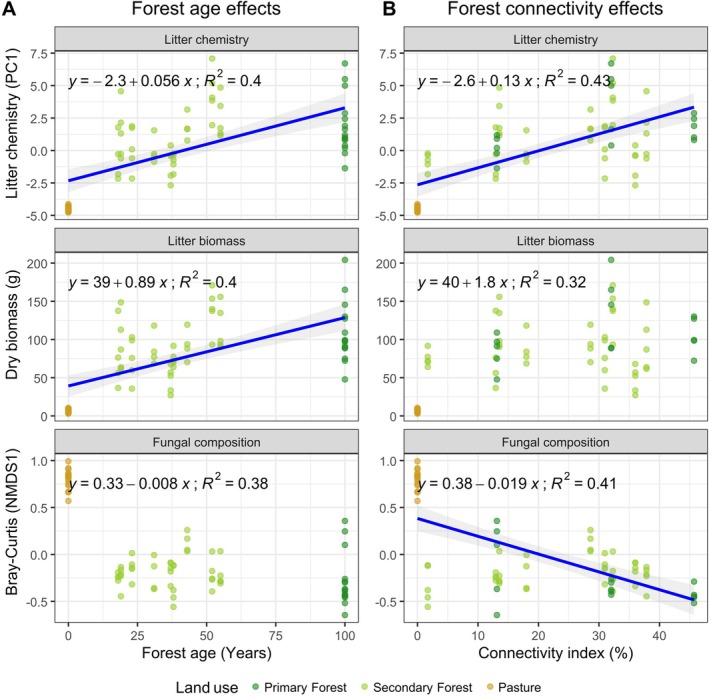
The effects of forest age (A) and forest connectivity (B) on litter chemistry, litter biomass, and litter fungal composition. Litter chemistry is represented by the first axis of the PCA. Litter fungal composition is represented by the first axis of the NMDS based on the Bray–Curtis dissimilarity. Lines represent significant relationships between variables. *R* ^2^ values represent the coefficient of correlation between variables.

Forest age and forest connectivity significantly explained the variation in litter chemistry, litter biomass, and fungal community composition (Figure 2; Table S3). Both forest age and connectivity were positively associated with the first principal component (PC1), which represented a gradient in litter chemistry. Litter chemistry was more strongly predicted by forest connectivity (*β* = 0.090, *p* = 0.010) than forest age (*β* = 0.040, *p* = 0.018). Conversely, litter biomass was significantly predicted by forest age (*β* = 0.650, *p* = 0.011), while the effect of forest connectivity was marginal (*β* = 1.060, *p* = 0.054). Fungal community composition, represented by the first axis of the NMDS, was significantly predicted by both forest age and connectivity, showing negative relationships (*β* = −0.005, *p* = 0.043; and *β* = −0.010, *p* = 0.042, respectively).

**FIGURE 3:**
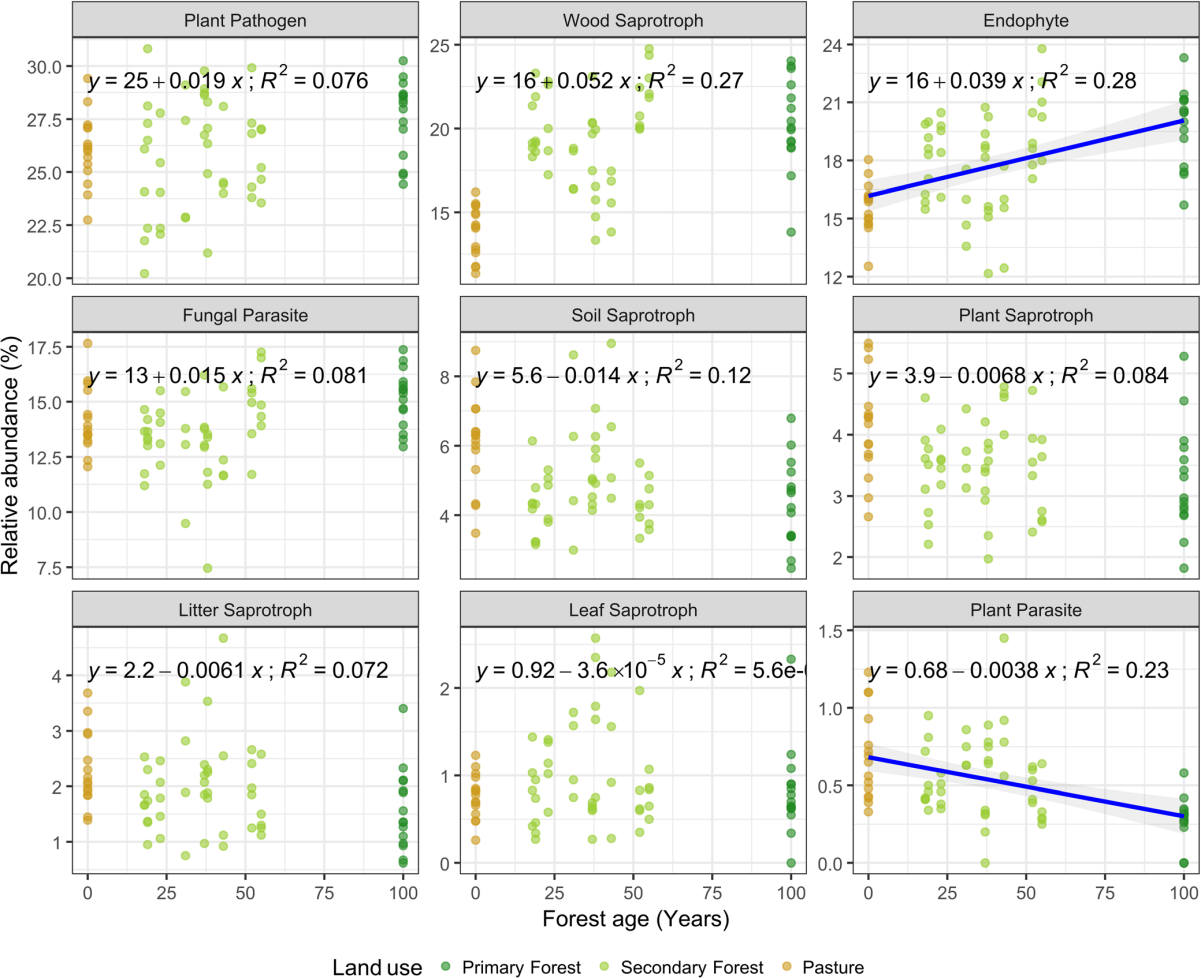
The effect of forest age on the relative abundance of fungal guilds during forest restoration. Lines represent significant relationships between fungal guilds and forest age and forest connectivity. *R* ^2^ values represent the coefficient of correlation between variables.

**FIGURE 4:**
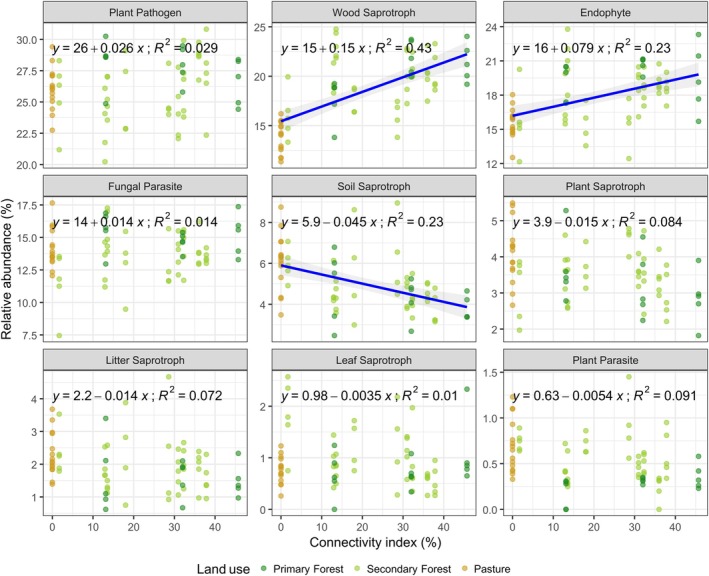
The effect of forest connectivity on the relative abundance of fungal guilds during forest restoration. Lines represent the relationships between fungal guilds and forest age and forest connectivity. *R* ^2^ values represent the coefficient of correlation between variables.

Forest age also significantly influenced the abundance of specific fungal guilds in the litter layer (Figure 3; Table S4). Specifically, it was positively associated with endophytes (*β* = 0.030, *p* = 0.049) and had a marginal negative association with plant parasites (*β* = 0.0034, *p* = 0.062). Forest connectivity further shaped fungal functional composition (Figure 4; Table S4), showing significant positive effects on wood saprotrophs (*β* = 0.130, *p* = 0.002) and marginal effects on endophytes (*β* = 0.060, *p* = 0.075). In contrast, soil saprotrophs were negatively associated with forest connectivity (*β* = −0.0084, *p* = 0.002).

### 3.4 Environmental Drivers of Fungal Guilds

Fungal guilds in leaf litter were significantly correlated with tree diversity, litter biomass, and nutrient content (Figure S6). Wood saprotrophs and endophytes showed positive correlations with most environmental variables, except with δ^13^C. In contrast, plant saprotrophs, soil saprotrophs, litter saprotrophs, and plant parasites were negatively correlated with these environmental variables. The strongest positive correlations were observed between wood saprotrophs and litter δ^15^N (*ρ* = 0.74), and between endophytes and litter δ^15^N (*ρ* = 0.72). Additionally, litter P and tree diversity were strongly correlated with wood saprotrophs abundance (both *ρ* = 0.65), while litter S and litter biomass were strongly associated with endophyte abundance (*ρ* = 0.53 and 0.50, respectively). Further, the strongest negative correlations included plant parasites and δ^15^N (*ρ* = −0.68), as well as soil saprotrophs and tree diversity (*ρ* = −0.44).

**FIGURE 5:**
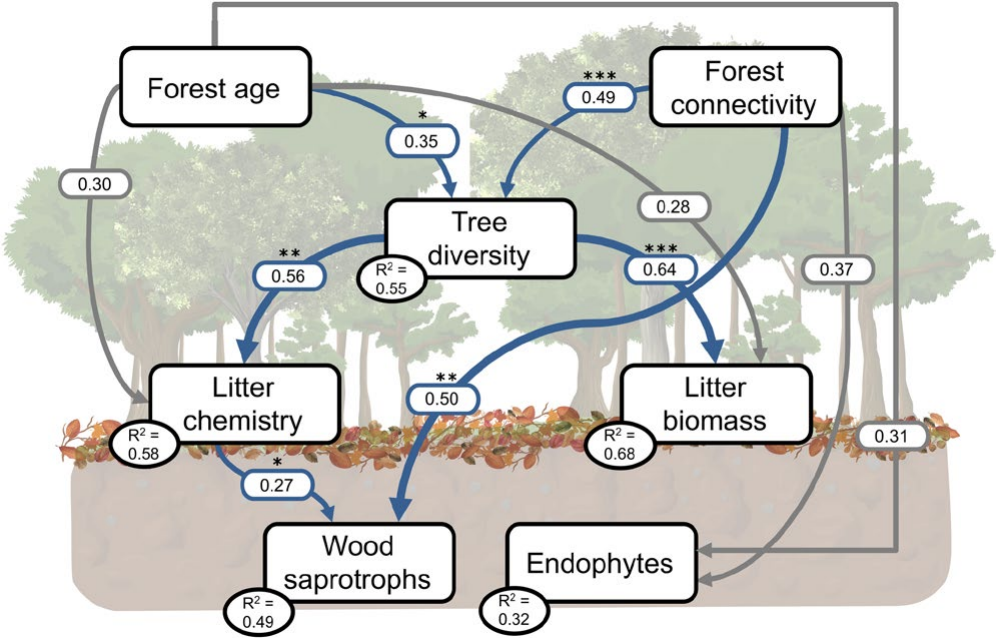
Structural Equation Model (SEM) shows the effects of forest age and connectivity during restoration on litter biomass, litter chemistry and litter fungal guilds. Arrows show the standardized path coefficient of linear regressions presented in Table S2. Blue arrow represents significant and positive relationships. Grey arrow represents non‐significant relationships. Asterisks indicate the significance level of each path (*p < 0.05; **p < 0.01; and ***p < 0.001). The SEM fit the data well, represented by the global goodness‐of‐fit with Fischer's C = 12.803; *p* = 0.687; df = 16; *n* = 75. The variation explained in each variable (*R* ^2^) represents the marginal response and is shown inside each box.

Structural equation modelling (SEM) showed that tree diversity was more strongly affected by forest connectivity (standardized path coefficient = 0.49) than by forest age (0.35) (Figure 5; Table S5). Tree diversity, in turn, had significant positive effects on both litter biomass (0.64) and litter chemistry (0.56), whereas forest age did not show a significant direct effect on either variable. Regarding fungal guilds, wood saprotrophs were positively affected by both forest connectivity (0.50) and litter chemistry (0.27). Endophyte abundance was marginally affected by forest connectivity (0.37) but not significantly influenced by forest age (0.31).

## 4 Discussion

In this study, we investigated the influence of forest age and connectivity on the fungal composition and functioning in forest litter during the restoration of Atlantic Forest fragments in Brazil. Our findings revealed that forest connectivity has moderate yet significant effects on fungal composition and litter chemistry. Specifically, forest connectivity accelerates the functional recovery of microbial decomposers by enhancing tree diversity and enriching the litter layer, strengthening nutrient cycling and supporting forest restoration. These findings challenge traditional restoration approaches that emphasize time since restoration as the primary determinant of ecosystem recovery. Instead, our results suggest that younger, well‐connected forest fragments may regain key functional services more rapidly than older but isolated fragments. These connections provide valuable insights into how litter microbes help restore ecosystem functions in lands previously degraded by grazing, from compacted, nutrient‐poor, and nearly bare soils to healthy forested ecosystems.

### 4.1 How Does Forest Age and Connectivity Affect Litter Attributes and Fungal Composition?

In our study, both forest connectivity and forest age correlated well with litter chemistry. However, only forest age had an indirect effect on litter biomass by enhancing tree diversity. This suggests that restored tropical forests progressively enhance tree leaf biomass over time, resulting in more litterfall and an accumulation of litter biomass (Souza et al. 2019). In contrast, older and connected secondary forests typically have higher tree diversity compared to older and isolated secondary forests (Rother et al. 2023), which affects the overall composition of litter chemistry and accumulation of litter biomass. This pattern likely occurs because forests with high tree diversity produce litter with a wider variety of complex carbon compounds, which in turn shapes the fungal community by promoting mutualistic interactions that enhance decomposition efficiency (García‐Palacios et al. 2013; Pei et al. 2017). However, it is important to note that our study was conducted in a single Atlantic Forest region and at a single sampling time point. Inclusion of additional regions and year‐round sampling would provide a more comprehensive representation of ecosystem dynamics.

In the context of forest restoration, a higher percentage of surrounding forest cover promotes species conservation and increases the quality of natural regeneration (Chazdon et al. 2025). Furthermore, greater connectivity may accelerate litter decomposition and increase nutrient release into the soil because diverse microbial communities, particularly fungi, are more efficient at breaking down organic matter than simplified ones (Buresova et al. 2019). Although we did not use litter bags to measure the decomposition process over time, we observed an increase in many fungal taxa related to litter degradation. Moreover, litter decomposition is a collaborative process involving insects, soil fauna and other microorganisms, and integrating the entire soil food web would provide a more holistic understanding of the specific contributions of litter fungi to decomposition processes.

Additionally, our results showed that the Fabaceae family was the most abundant and diverse in secondary forests (Table S5), suggesting that a significant portion of litter N and P may originate from these species. For example, Fabaceae trees are fast‐growing and capable of biological N fixation, producing high‐protein litter with elevated N and P concentrations (Neves et al. 2022; Wang et al. 2022). We also found that litter N and P content were positively correlated with fungal guilds such as endophytes and wood saprotrophs. This indicates that litter nutrient availability is a key driver of fungal community shifts, altering the predominance of oligotrophic Ascomycota to copiotrophic Basidiomycota (Guo et al. 2024). This microbial transition might largely induce by changes in tree species composition (Urbanová et al. 2015) that further affect environmental factors such as soil moisture, pH and nutrient availability (Huang et al. 2023).

We also observed a shift from oligotrophic to copiotrophic fungi in our study. Pasture litter was dominated by Ascomycota and had a low abundance of Basidiomycota, whereas primary forest litter showed the opposite trend, with secondary forests showing intermediate values (Figure S2). These transitions likely reflect changes in litter quality and decomposition stages during forest succession. In the early successional stages, litter is typically rich in cellulose but poor in nutrients, favouring Ascomycota. In contrast, more mature forests produce litter richer in lignin and nutrients, which supports Basidiomycota (Baldrian 2017; Hannula et al. 2017). This Ascomycota‐to‐Basidiomycota ratio is a strong indicator of forest successional stage (Gourmelon et al. 2016). In our case, forest connectivity was directly associated with litter chemistry and fungal composition compared to forest age, suggesting that higher percentages of forest cover enhance nutrient concentration in litter, further shaping the fungal composition. Together, these effects alter the fungal community from oligotrophic to copiotrophic taxa, which potentially may lead to faster decomposition and nutrient cycling. We conclude that forest connectivity enhanced the shift from oligotrophic to copiotrophic fungi, likely reflecting a better restoration of litter decomposition processes.

### 4.2 Forest Connectivity and Its Relationship With Saprophytic and Endophytic Communities

It is well established that increasing connectivity between forest fragments enhances the diversity and functionality of plants and macrofauna during forest restoration (Rother et al. 2018; Matos et al. 2020). In this study, we demonstrate that the effects of forest age and connectivity also occur at smaller scales, particularly in litter chemistry and fungal communities. These effects become more pronounced at intermediate and late stages of restoration compared to the earliest stages. While fungal composition is directly linked to litter chemistry and host specificity (Zhang et al. 2020), tree composition is more strongly associated with broader environmental variables such as forest connectivity and regional edaphic conditions (Zucchi et al. 2018). This creates an indirect pathway of forest connectivity influencing fungal composition in the litter layer.

Endophytic and pathogenic fungi in fresh leaves depend on carbon supplied by the host plant (Vincent et al. 2016; Zhang et al. 2020), but it is suggested that many groups continue their life cycle in the leaf litter after senescence (Otsing et al. 2018). Some studies further suggest that endophytes remain an active and substantial part of the litter fungal community even 1 year after litterfall (Guerreiro et al. 2018). At the same time, many endophytic fungi shift to a saprophytic lifestyle, becoming part of the initial decomposer community and influencing the succession of later fungal groups adapted to degrading more recalcitrant compounds, such as lignin (Kirker et al. 2020). Here, endophytic fungi and litter biomass were mainly associated with forest age, suggesting that litter accumulation in older forests is more related to fresh litterfall. In contrast, saprotrophs and litter chemistry were more strongly associated with forest connectivity, implying an association with older, more decomposed litter characterized by higher C/N ratios and greater lignin content (Leifheit et al. 2024). This interpretation is further supported by the observed shift in the Ascomycota‐to‐Basidiomycota ratio in our litter samples, with Basidiomycota becoming more dominant in later stages of litter decomposition.

Specifically, Sordariomycetes (Ascomycota) and Agaricomycetes (Basidiomycota) are important groups of decomposers of complex substrates such as lignin and tannin (Veen et al. 2021), and were more abundant in our old‐growth forests. In contrast, Dothideomycetes (Ascomycota) was a biomarker for pasture litter, previously shown to be strongly associated with higher cellulose content in litter (Veen et al. 2021). Interestingly, both Sordariomycetes and Dothideomycetes are cosmopolitan and highly versatile, capable of functioning as either saprophytes or endophytes, depending on resource availability (Freitas et al. 2021). These functional adaptations contribute to fungal multifunctionality and niche specialization in the litter layer (Bastida et al. 2016; Bhatnagar et al. 2018). Moreover, many wood saprotrophs belong to the class Agaricomycetes and are considered essential for breaking down recalcitrant organic matter, representing a good indicator of forest management quality (Asplund et al. 2026). The identification of these taxa in the litter layer helps explain the transition from degraded forests to more efficient nutrient‐cycling systems, even during climate‐stress events (Xu et al. 2025; Zhang et al. 2026).

Furthermore, litter isotope signature (*δ* ^13^C and *δ* ^15^N) was significantly correlated with both forest age and forest connectivity, as well as with most fungal guilds with in the litter. On one hand, saprophytes and endophytes were associated with higher *δ* ^15^N values, while plant parasites were associated with lower *δ* ^13^C values. This suggests that microbes preferentially consume lighter isotopes (^12^C and ^14^N) during decomposition, resulting in an enrichment of heavier isotopes in the remaining litter (Bieluczyk, Asselta, et al. 2023). *δ* ^15^N enrichment is often attributed to both microbial biomass incorporation into decomposing litter (Bieluczyk, Merloti, et al. 2023), and increased biological nitrogen fixation (Zanini et al. 2021). On the other hand, the relationships between litter isotopic signature and forest age and connectivity are likely influenced by the origin and decomposition stage of organic matter. For instance, higher *δ* ^13^C values are often derived from C_4_ plants and are indicative of early‐stages restoration (Zanini et al. 2021), but they can also reflect older, more recalcitrant litter, as heavier isotopes form stronger chemical bonds that are more resistant to microbial breakdown, resulting in higher *δ* ^13^C (Xiong et al. 2020). Although no strong evidence directly links forest connectivity to litter isotope signature, we speculate that the enrichment of *δ* ^15^N in more connected forests may result from both the presence of nitrogen‐fixing bacteria, which supply additional N to other decomposer microorganisms, and by the accumulation of *δ* ^15^N in recalcitrant organic matter.

## 5 Conclusions

Increased forest connectivity led to higher proportions of wood saprotrophs and endophytes in the litter layer, though this connectivity effect was more evident in the intermediate and late stages of forest succession. Secondary forests with at least 30% connectivity were high in wood saprotrophs and endophytes in the litter layer, and these proportions increased along the chronosequence of forest restoration as connectivity increased. Furthermore, the presence of key species such as Sordariomycetes and Agaricomycetes (Basidiomycota) in the litter layer is a good indicator of restored forest. Our findings suggest that enhancing forest connectivity is a promising measure for restoring the fungal litter community, especially in intermediate and late stages of forest restoration. Although these restoration stages had a higher abundance of these fungal guilds compared to early secondary forests, enhancing connectivity can benefit all stages of restoration.

The litter layer responds well to both forest age and connectivity, making it a useful metric for assessing ecosystem services in secondary forests, such as promoting fungal diversity and supporting carbon sequestration and storage through microbial activity. Whether a loss of forest connectivity leads to declines in fungal communities and reduced nutrient cycling, potentially contributing to forest degradation through proliferation of more plant parasites and pathogens, requires further studies. Investigating these effects is relevant, as it may explain how climate change effects may exacerbate future declines in plant diversity and alter community composition. Our study shows that these processes may, at least in part, be reversed by restoring forests while considering spatial connectivity of the new and existing forest areas. Future studies incorporating metatranscriptomics would provide valuable insights into which fungal genes are actively expressed and which decomposition pathways are functioning in real time, enabling a more mechanistic understanding of fungal contribution to forest restoration. While the practical implications of increasing connectivity on microbial attributes may seem minor, these changes can increase the resilience of newly restored forests in the face of future climate change scenarios when considered at a broader scale and over the long term. The One Health concept recognizes that human, animal and plant health are closely linked to environmental and ecosystem stability.

## Author Contributions

**Guilherme Lucio Martins:** conceptualization, formal analysis, investigation, methodology, visualization, writing – original draft; **Dina in 't Zandt:** conceptualization, formal analysis, supervision, writing – review and editing; **Gabriel Silvestre Rocha:** conceptualization, investigation, validation, writing – review and editing; **Luis Fernando Merloti:** conceptualization, investigation, data curation, writing – review and editing; **Wanderlei Bieluczyk:** conceptualization, investigation, validation, writing – review and editing; **Robert Timmers:** conceptualization, data curation, writing – review and editing; **Ricardo Ribeiro Rodrigues:** data curation, writing – review and editing; **Siu Mui Tsai:** conceptualization, funding acquisition, project administration, resources, supervision, writing – review and editing; **Wim H. van der Putten:** conceptualization, funding acquisition, resources, supervision, writing – review and editing. The authors declare that they have obtained all the appropriate licences and permissions for the sites studied.

## Funding

This work was supported by Fundação de Amparo à Pesquisa do Estado de São Paulo (2018/19000‐4, 2018/19000‐6, 2022/05561‐0, Process 2021/00976‐4, 2023/18333‐8). HORIZON EUROPE Marie Sklodowska‐Curie Actions (101110604).

## Conflicts of Interest

The authors declare no conflicts of interest.

## Supporting information


**Table S1:** Geographic coordinates of the studied sites.
**Figure S1:** Conceptual Structural Equation Model (SEM). See Table S2 for hypothesis on individual relationships.
**Table S2:** Hypothesized relationships between the variables used in the Structural Equation Model (SEM).
**Figure S2:** (A) Relative abundance of litter fungal community at phylum level. (B) Relative abundance of litter fungal community at class level. Different letters indicate differences according to the Tukey‐HSD test (*p* < 0.05).
**Figure S3:** Random Forest analysis shows fungal biomarkers in leaf litter at the class level for each land‐use type. Asterisks indicate significant differences among land‐use types (*p < 0.05; **p < 0.01; ***p < 0.001).
**Figure S4:** The relative abundance of functional fungal guilds in different land use types. Letters represent significant differences according to the Tukey‐HSD test (*p* < 0.05).
**Figure S5:** Litter attributes in different land‐use types. Letters represent significant differences according to the Tukey‐HSD test (*p* < 0.05).
**Table S3:** Summary of linear mixed effect model showing the relationships of forest age and forest connectivity on litter attributes.
**Table S4:** Summary of linear mixed effect models showing the effects of forest age and forest connectivity on the relative abundance of litter fungal guilds.
**Figure S6:** Spearman correlation between the litter fungal guilds and the environmental variables. Coloured tiles represent significant correlations (*p* < 0.01).
**Table S5:** Full summary of the Structural Equation Model (SEM) used in the study.
**Table S6:** Plants species composition in all studied sites.

## Data Availability

The datasets and R code generated during and/or analysed during the current study are available in the Zenodo repository: 10.5281/zenodo.17100518. The sequences are available at the NCBI Sequence Read Archive under the identification PRJNA1296929.
