# Supplementary material for: Connectivity and Age of Restored Atlantic Forest Fragments Drives Composition and Functionality of the Fungal Community in the Leaf Litter Layer
Source: Mol Ecol. 2026 Mar 24;35(6):e70325. doi: 10.1111/mec.70325 (PMC13010786; doi:10.1111/mec.70325)
Supplement: Supplementary file 1 — Table S1: Geographic coordinates of the studied sites. Figure S1: Conceptual Structural Equation Model (SEM). See Table S2 for hypothesis on individual relationships. Table S2: Hypothesized relationships between the variables used in the Structural Equation Model (SEM). Figure S2: (A) Relative abundance of litter fungal community at phylum level. (B) Relative abundance of litter fungal community at class level. Different letters indicate differences according to the Tukey‐HSD test (p < 0.05). Figure S3: Random Forest analysis shows fungal biomarkers in leaf litter at the class level for each land‐use type. Asterisks indicate significant differences among land‐use types (*p < 0.05; **p < 0.01; ***p < 0.001). Figure S4: The relative abundance of functional fungal guilds in different land use types. Letters represent significant differences according to the Tukey‐HSD test (p < 0.05). Figure S5: Litter attributes in different land‐use types. Letters represent significant differences according to the Tukey‐HSD test (p < 0.05). Table S3: Summary of linear mixed effect model showing the relationships of forest age and forest connectivity on litter attributes. Table S4: Summary of linear mixed effect models showing the effects of forest age and forest connectivity on the relative abundance of litter fungal guilds. Figure S6: Spearman correlation between the litter fungal guilds and the environmental variables. Coloured tiles represent significant correlations (p < 0.01). Table S5: Full summary of the Structural Equation Model (SEM) used in the study. Table S6: Plants species composition in all studied sites. [file MEC-35-e70325-s001.docx]

**Supplementary information**

**Connectivity and age of restored Atlantic Forest fragments drives composition and functionality of the fungal community in the leaf litter layer**

Guilherme Lucio Martins ^1,2,3*^; Dina in 't Zandt ^3,4^; Luis Fernando Merloti ^1,2,3^; Wanderlei Bieluczyk ^1^; Gabriel Silvestre Rocha ^1,4^; Robert Timmers ^5^; Ricardo Ribeiro Rodrigues ^2^; Siu Mui Tsai ^1^; Wim H. van der Putten ^3,6^

^1^ Center for Nuclear Energy in Agriculture (CENA), University of São Paulo (USP), Piracicaba, SP, Brazil

^2^ Luiz de Queiroz College of Agriculture (ESALQ), University of São Paulo (USP), Piracicaba, SP, Brazil

^3^ Department of Terrestrial Ecology, Netherlands Institute of Ecology (NIOO-KNAW), Wageningen, The Netherlands

^4^ Department of Ecology, Radboud Institute for Biological and Environmental Sciences (RIBES), Radboud University, Nijmegen, The Netherlands

^5^ Department of Microbial Ecology, Netherlands Institute of Ecology (NIOO-KNAW), Wageningen, The Netherlands

^6^ Ecology & Biodiversity Group, Department of Biology, Utrecht University, Utrecht, The Netherlands

^7^ Department of Nematology, Wageningen University & Research, Wageningen, The Netherlands

^*^ Corresponding author: guilhermelucio@usp.br / g.luciomartins@nioo.knaw.nl

**Table S1.** Geographic coordinates of the studied sites.

| **Land use** | **Plot age** | **Age class** | **Connectivity^*^** | **Connectivity class** | **Lat** | **Log** | **Site description** |
| --- | --- | --- | --- | --- | --- | --- | --- |
|  |  |  | 45.71 | High | -22.138136 | -47.847577 |  |
| Primary Forest | > 100 | Old-growth | 32.02 | Mid | -22.923611 | -47.661718 | Old growth conserved forest |
|  |  |  | 13.13 | Low | -22.37757 | -47.321191 |  |
|  | 55 |  | 13.53 | Low | -22.288948 | -47.623007 |  |
|  | 52 | Late | 32.29 | High | -22.33904572 | -47.57296578 | Passive restoration former pasturelands |
|  | 43 |  | 28.60 | Mid | -22.23692377 | -47.59399561 |  |
|  | 38 |  | 1.70 | Low | -22.29307595 | -47.59562467 |  |
| Secondary Forest | 37 | Intermediate | 36.00 | High | -22.3460252 | -47.57490316 | Passive restoration former pasturelands |
|  | 31 |  | 18.01 | Mid | -22.21502106 | -47.59199846 |  |
|  | 23 |  | 30.97 | Mid | -22.330099 | -47.557035 |  |
|  | 19 | Early | 37.85 | High | -22.34200863 | -47.57337753 | Passive restoration former pasturelands |
|  | 18 |  | 12.94 | Low | -22.35734708 | -47.56312814 |  |
|  |  |  | 0.00 |  | -22.3469444 | -47.5738889 |  |
| Pasture | 0 | – | 0.00 | – | -22.2891667 | -47.6191667 | Pasture for cattle grazing |
|  |  |  | 0.00 |  | -22.2919444 | -47.5972222 |  |

^*^ = Forest connectivity was calculated as the percentage of native forest cover (native or planted) within a 1 km radius around the plot using satellite images.

**
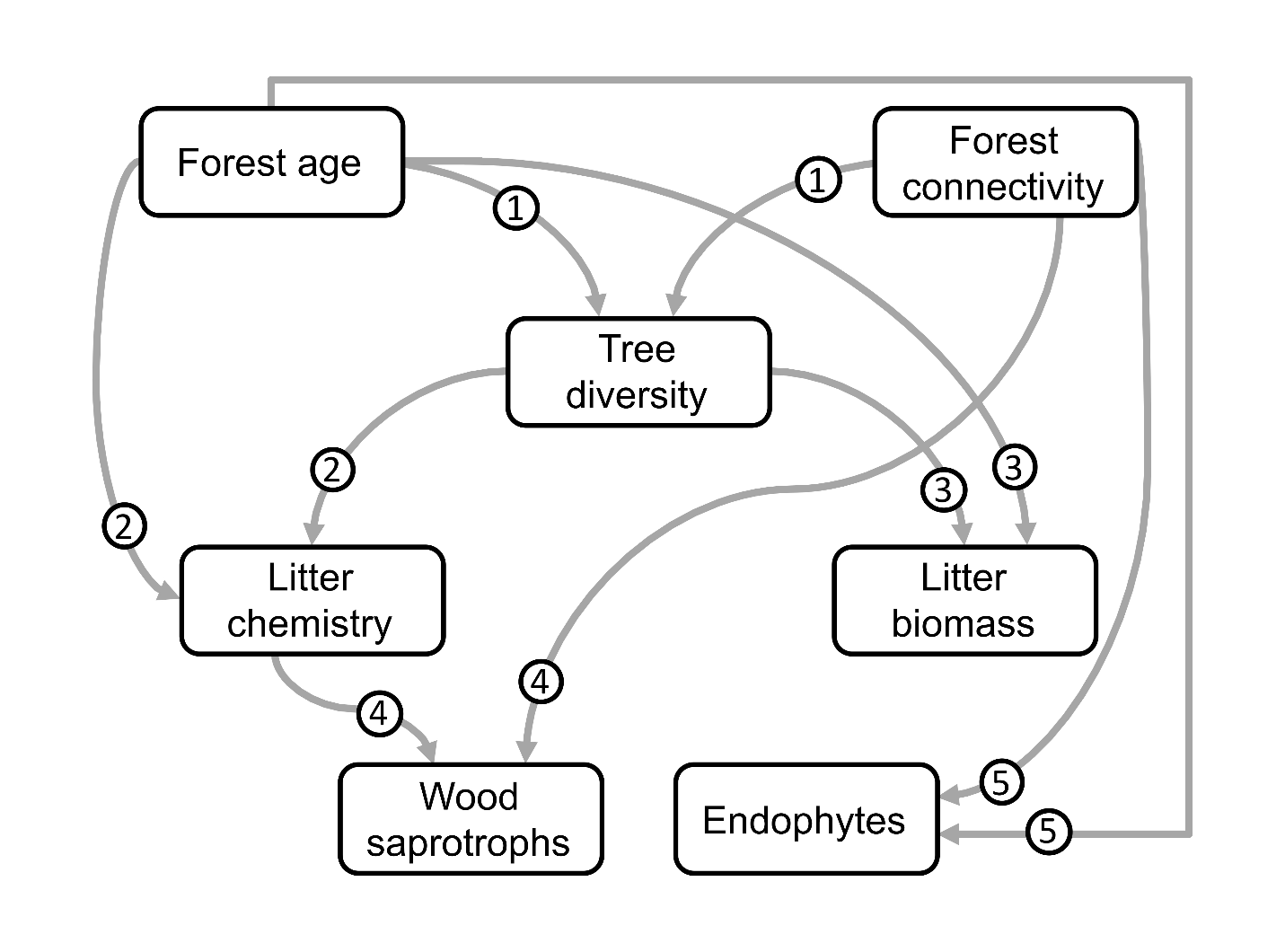
**

**Figure S1.** Conceptual Structural Equation Model (SEM). See supplementary Table S2 for hypothesis on individual relationships.

**Table S2.** Hypothesized relationships between the variables used in the Structural Equation Model (SEM).

| **Path** | **Reason** | **Reference** |
| --- | --- | --- |
| 1. Tree diversity ~ Forest age + Forest connectivity | Older and more connected forests are expected to have a higher diversity of tree species than low-connected forests. This is due to an equitable abundance of species, reflecting balanced ecological succession in these areas. Over time, local changes through restoration may increase animal and plant biodiversity, as well as pollination services. | (César et al., 2021; Garcia et al., 2016; Rother et al., 2018) |
| 1. Litter chemistry ~ Tree diversity + Forest age | The chemical composition of litter, *i.e.*, its elemental composition, is expected to change over time as restoration progresses. Higher plant diversity may affect litter chemistry through restoration time due to an increased input of substrates and different decomposition rates, which can result in higher levels of macronutrients and micronutrients in later stages of forest restoration. | (Getaneh et al., 2022; Lanuza et al., 2019; Zhang et al., 2022) |
| 1. Litter biomass ~ Tree diversity + Forest age | Due to higher tree biomass and consequently higher litterfall, leaf litter accumulation is expected to increase with forest age. Additionally, forests with higher plant diversity are expected to have higher litterfall due to different C:N ratios and the presence of more substrates with lower decomposition rates. | (Bieluczyk et al., 2025; Horodecki et al., 2019; Schnabel et al., 2025) |
| 1. Wood saprotrophs ~ Litter chemistry + Forest connectivity | Recent studies show that the ecology of some fungal groups depends on spatial scale as well as other environmental factors, such as vegetation, climate, and litter chemistry. Older and more connected forests are expected to have a higher abundance of saprotrophs due to the dominance of tree species and the higher content of lignin and substrates with high C:N ratios. | (Beidler & Pritchard, 2017; Odriozola et al., 2023; Urbanová et al., 2015) |
| 1. Endophytes ~ Forest age + Forest connectivity | Similarly, the abundance of endophytes is expected to depend on the dominant tree species and spatial scale. Older forests with lower connectivity are expected to have a higher abundance of endophytes because trees have fewer resources and need microbial help to absorb nutrients and protect themselves against environmental stress. | (Barbi et al., 2025; Hannula et al., 2017; Yang et al., 2022) |


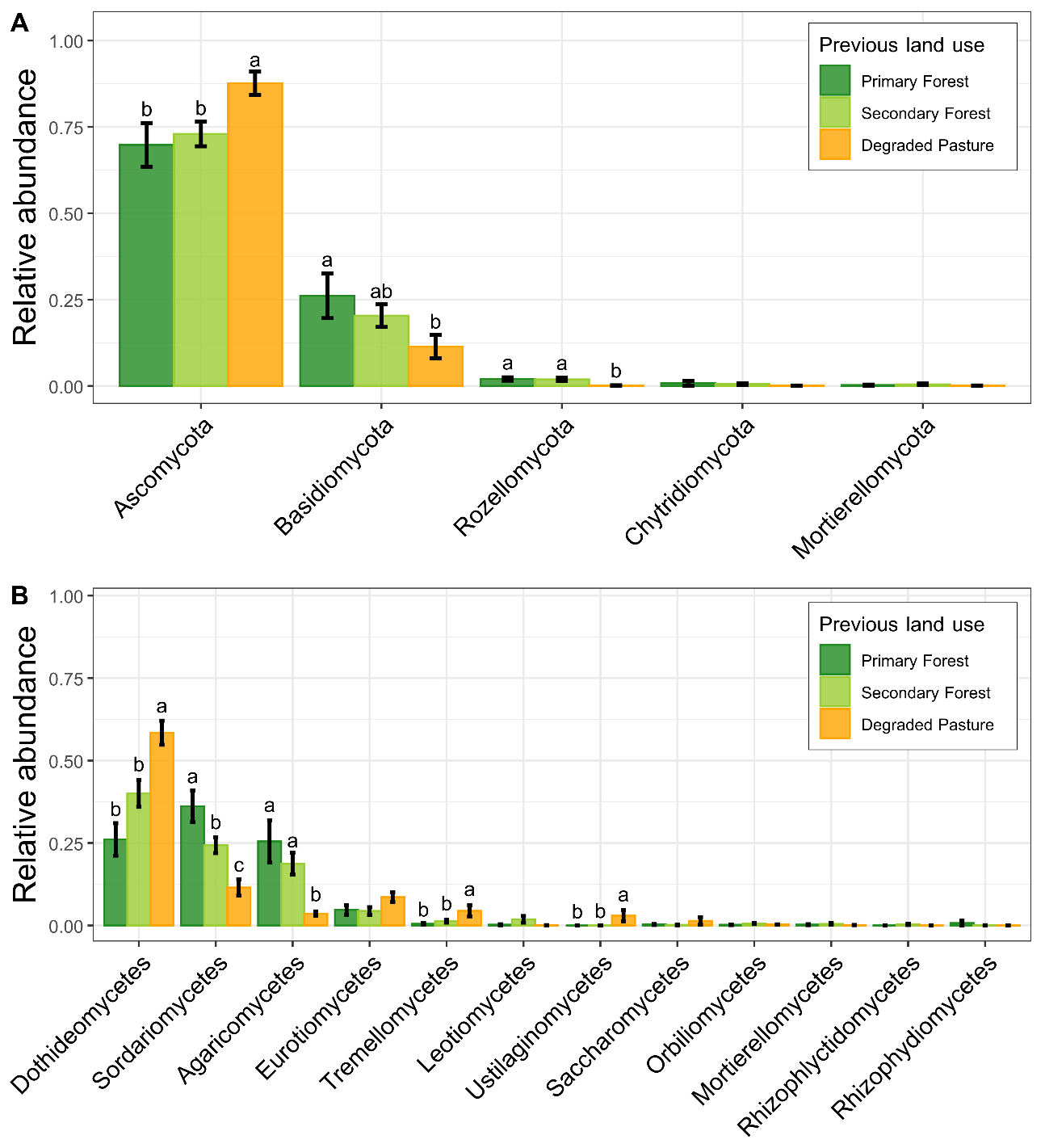


**Figure S2.** (A) Relative abundance of litter fungal community at phylum level. (B) Relative abundance of litter fungal community at class level. Different letters indicate differences according to the Tukey-HSD test (p < 0.05).

**
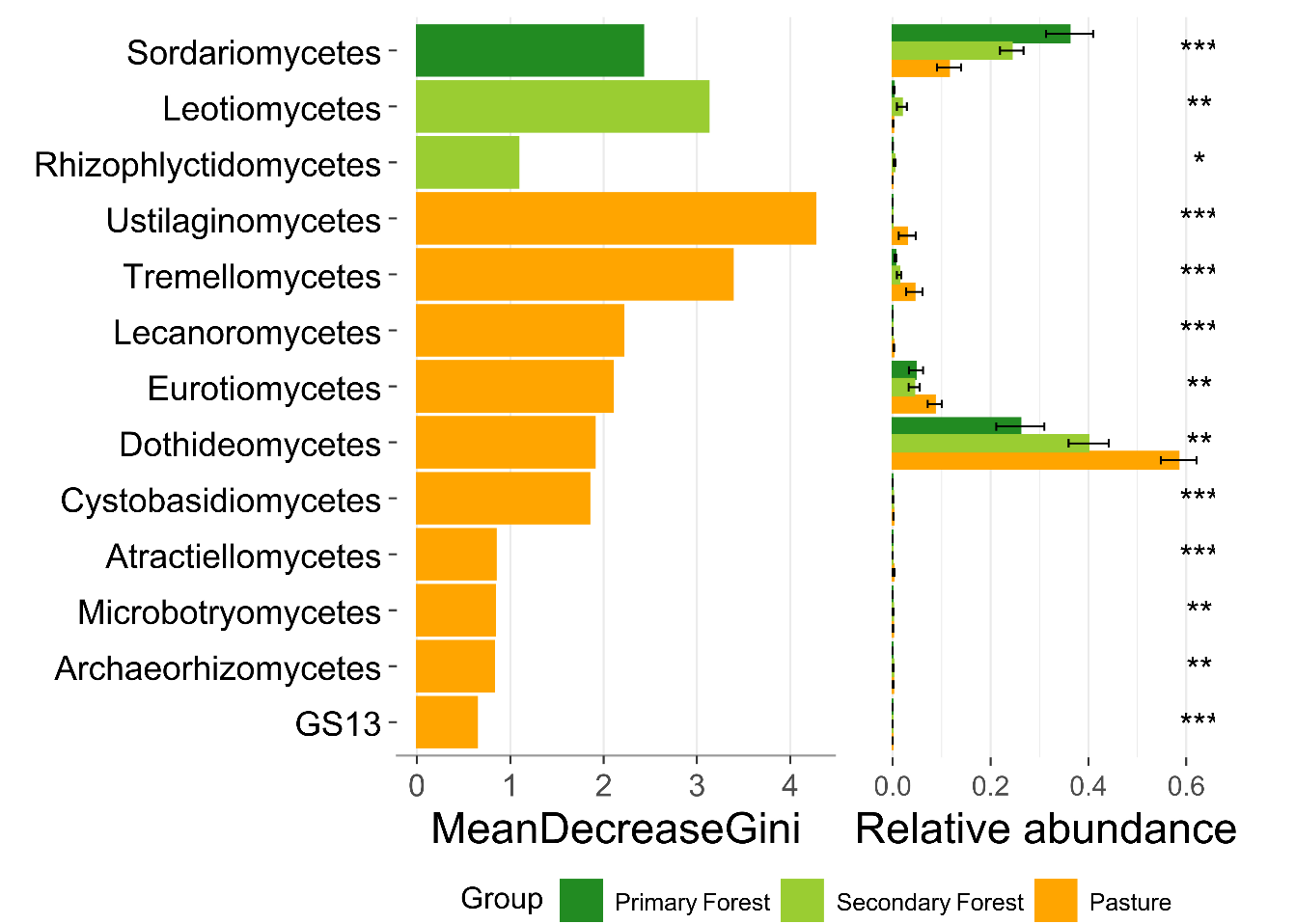
**

**Figure S3.** Random Forest analysis shows fungal biomarkers in leaf litter at the class level for each land-use type. Asterisks indicate significant differences among land-use types (* = p < 0.05; ** = p < 0.01; *** = p < 0.001).


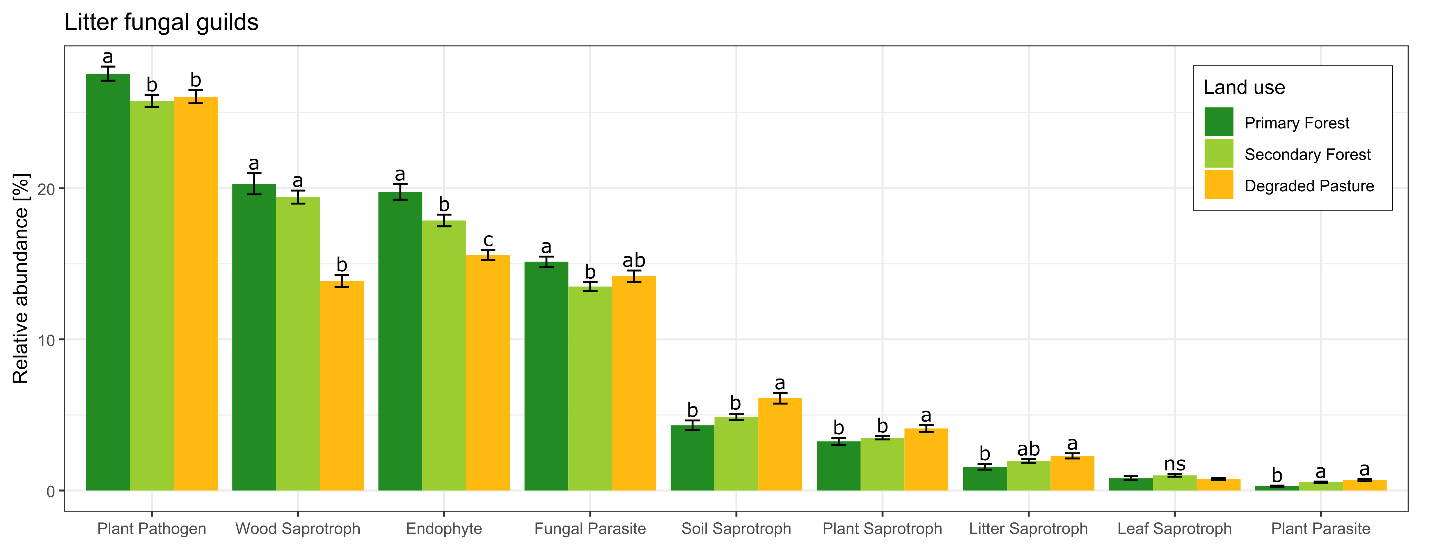


**Figure S4.** The relative abundance of functional fungal guilds in different land use types. Letters represent significant differences according to the Tukey-HSD test (*p* < 0.05).

**
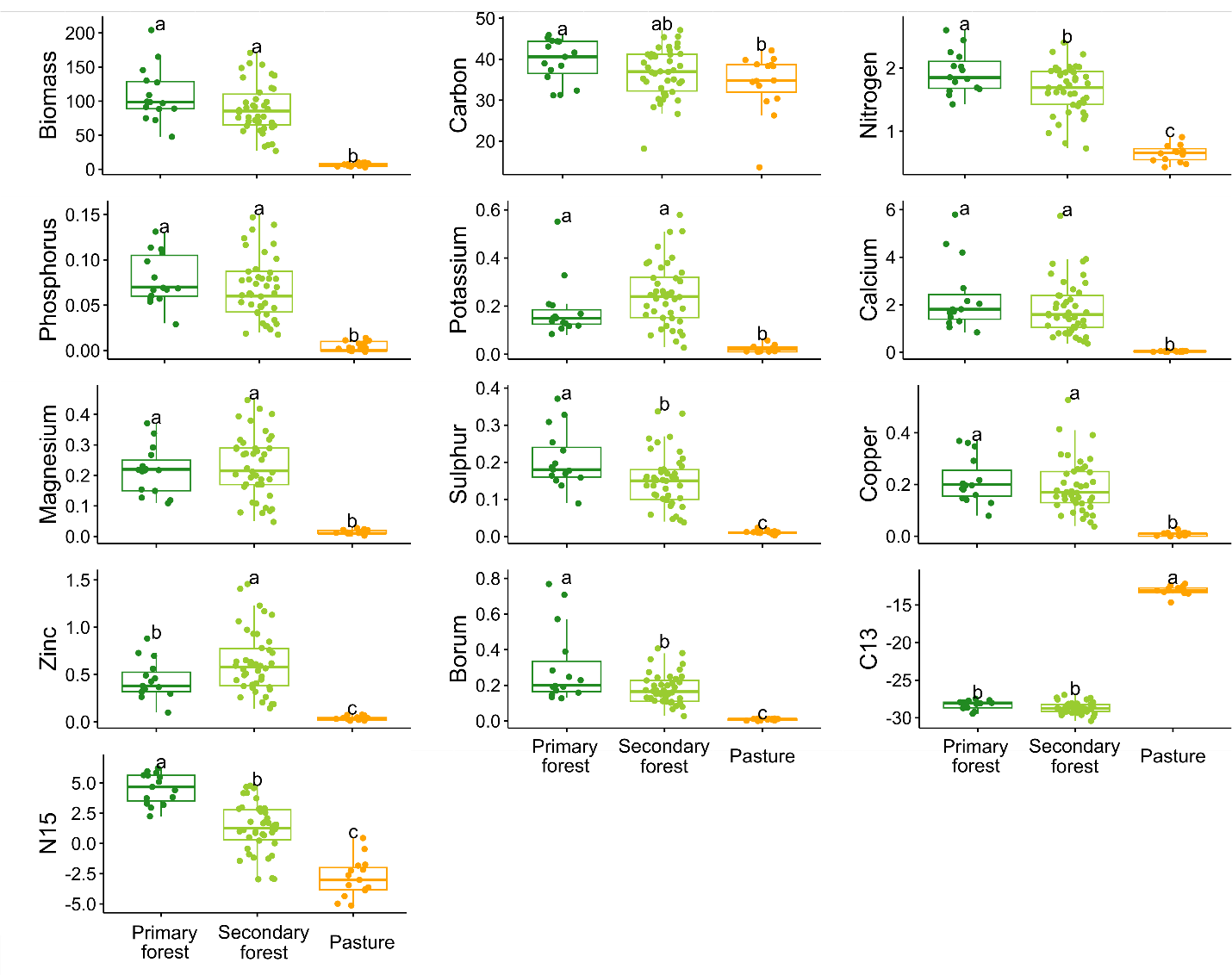
**

**Figure S5.** Litter attributes in different land-use types. Letters represent significant differences according to the Tukey-HSD test (p < 0.05).

**Table S3.** Summary of linear mixed effect model showing the relationships of forest age and forest connectivity on litter attributes.

| **Response** | **Predictor** | **Coefficient** | **SE** | **95% CI** | **t-value** | **p-value** | **R2m** | **R2c** |
| --- | --- | --- | --- | --- | --- | --- | --- | --- |
| Litter chemistry | Forest age | 0.040 | 0.020 | [0.01, 0.07] | 2.42 | **0.018** | 0.521 | 0.795 |
|  | Forest connectivity | 0.090 | 0.030 | [0.02, 0.15] | 2.64 | **0.010** |  |  |
| Litter biomass | Forest age | 0.650 | 0.250 | [0.15, 1.14] | 2.6 | **0.011** | 0.454 | 0.724 |
|  | Forest connectivity | 1.060 | 0.540 | [-0.02, 2.14] | 1.96 | 0.054 |  |  |
| Fungal composition | Forest age | -0.005 | 0.003 | [-0.01, 0.00] | -2.06 | **0.043** | 0.448 | 0.880 |
|  | Forest connectivity | -0.010 | 0.005 | [-0.02, 0.00] | -2.08 | **0.042** |  |  |

SE represents the standard error, 95% CI represents the 95% confidence interval, and R2m and R2c represent the model marginal and conditional effects, respectively. Bold numbers represent significant p-values.

**Table S4.** Summary of linear mixed effect models showing the effects of forest age and forest connectivity on the relative abundance of litter fungal guilds.

| **Response** | **Predictor** | **Coefficient** | **SE** | **95% CI** | **t-value** | **p-value** | **R2m** | **R2c** |
| --- | --- | --- | --- | --- | --- | --- | --- | --- |
| Plant Pathogen | Forest age | 0.0200 | 0.01 | [-0.01, 0.04] | 1.21 | 0.229 | 0.076 | 0.297 |
|  | Forest connectivity | 0.0100 | 0.03 | [-0.04, 0.07] | 0.46 | 0.649 |  |  |
| Wood Saprotroph | Forest age | 0.0200 | 0.02 | [-0.02, 0.06] | 1.08 | 0.285 | 0.456 | 0.763 |
|  | Forest connectivity | 0.1300 | 0.04 | [0.05, 0.21] | 3.26 | **0.002** |  |  |
| Endophyte | Forest age | 0.0300 | 0.01 | [0.00, 0.05] | 1.85 | **0.049** | 0.363 | 0.597 |
|  | Forest connectivity | 0.0600 | 0.03 | [-0.01, 0.12] | 1.81 | 0.075 |  |  |
| Fungal Parasite | Forest age | 0.0100 | 9.37E-03 | [-0.01, 0.03] | 1.46 | 0.149 | 0.074 | 0.250 |
|  | Forest connectivity | 0.0038 | 0.02 | [-0.04, 0.05] | 0.18 | 0.856 |  |  |
| Soil Saprotroph | Forest age | -0.0012 | 1.19E-03 | [0.00, 0.00] | -1.01 | 0.317 | 0.241 | 0.276 |
|  | Forest connectivity | -0.0084 | 2.67E-03 | [-0.01, 0.00] | -3.15 | **0.002** |  |  |
| Litter Saprotroph | Forest age | -0.0017 | 1.06E-03 | [0.00, 0.00] | -1.61 | 0.113 | 0.106 | 0.106 |
|  | Forest connectivity | -0.0030 | 2.39E-03 | [-0.01, 0.00] | -1.25 | 0.214 |  |  |
| Plant Saprotroph | Forest age | -0.0053 | 4.27E-03 | [-0.01, 0.00] | -1.23 | 0.223 | 0.096 | 0.283 |
|  | Forest connectivity | -0.0075 | 9.49E-03 | [-0.03, 0.01] | -0.79 | 0.435 |  |  |
| Leaf Saprotroph | Forest age | 0.0017 | 3.11E-03 | [0.00, 0.01] | 0.55 | 0.583 | 0.030 | 0.304 |
|  | Forest connectivity | -0.0073 | 6.89E-03 | [-0.02, 0.01] | -1.05 | 0.297 |  |  |
| Plant Parasite | Forest age | -0.0034 | 1.77E-03 | [-0.01, 0.00] | -1.9 | 0.062 | 0.207 | 0.623 |
|  | Forest connectivity | -0.0017 | 3.83E-03 | [-0.01, 0.01] | -0.46 | 0.650 |  |  |

SE represents the standard error, 95% CI represents the 95% confidence interval, and R2m and R2c represent the model marginal and conditional effects, respectively. Bold numbers represent significant p-values.


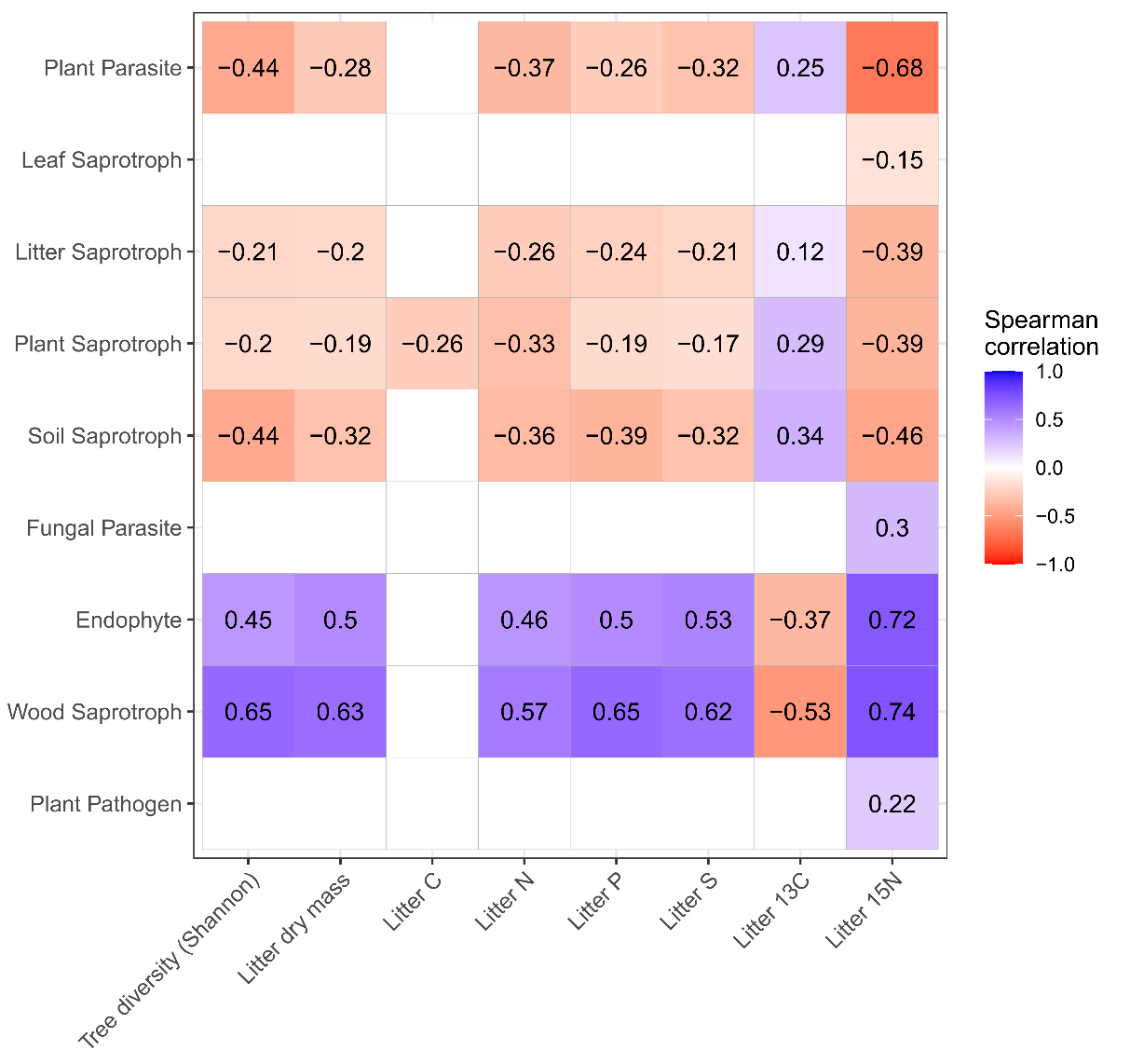


**Figure S6.** Spearman correlation between the litter fungal guilds and the environmental variables. Colored tiles represent significant correlations (p < 0.01).

**Table S5.** Full summary of the Structural Equation Model (SEM) used in the study.

| Response | Predictor | Estimate | Std.Error | *df* | Crit.Value | Std.Estimate | P.Value | Significance |
| --- | --- | --- | --- | --- | --- | --- | --- | --- |
| Tree diversity | Forest age | 0.0106 | 0.0048 | 13.0633 | 2.2166 | 0.3534 | 0.045 | * |
| Tree diversity | Connectivity | 0.0334 | 0.0022 | 17.5059 | 15.1027 | 0.4946 | 0.000 | *** |
| Litter chemistry | Tree diversity | 1.6507 | 0.4983 | 13.0859 | 3.3128 | 0.5594 | 0.006 | ** |
| Litter chemistry | Forest age | 0.0264 | 0.015 | 12.7304 | 1.7566 | 0.299 | 0.103 |  |
| Litter biomass | Tree diversity | 2.0569 | 0.4697 | 13.0602 | 4.3788 | 0.6382 | 0.001 | *** |
| Litter biomass | Forest age | 0.0269 | 0.0142 | 12.7157 | 1.8993 | 0.2789 | 0.080 |  |
| Wood Saprotroph | Litter chemistry | 0.3139 | 0.1412 | 64.5363 | 2.2237 | 0.2759 | 0.030 | * |
| Wood Saprotroph | Connectivity | 0.1126 | 0.0367 | 19.9566 | 3.068 | 0.4974 | 0.006 | ** |
| Endophyte | Forest age | 0.0013 | 8e-04 | 12.237 | 1.6146 | 0.3116 | 0.132 |  |
| Endophyte | Connectivity | 0.0035 | 0.0018 | 13.8504 | 1.979 | 0.3709 | 0.068 |  |
| ~~Litter biomass | ~~Litter chemistry | 0.9293 | - | 75 | 20.8947 | 0.9293 | 0.000 | *** |
| ~~Endophyte | ~~Wood Saprotroph | 0.5003 | - | 75 | 4.7995 | 0.5003 | 0.000 | *** |

Global goodness-of-fit shows Fisher's C = 12.803 with P-value = 0.687 and on 16 degrees of freedom (*df*). Akaike Information Criterion (AIC) shows a value of 594.87. Sample size is *n* = 75. Std.Estimate shows the standardized estimate values for path coefficients. Significance levels represents ‘* = *p* < 0.05’; ‘** = *p* < 0.01’; and ‘*** = *p* < 0.001’. Correlated errors used in the SEM are represented by ‘~~’ symbol.

**Table S6.** Plants species composition in all studied sites.

| Family | Species | Sites | | | | | | | | | | | |
| --- | --- | --- | --- | --- | --- | --- | --- | --- | --- | --- | --- | --- | --- |
|  |  | Early secondary forest | | | Intermediate secondary forest | | | Late secondary forest | | | Old-growth primary forest | | |
|  |  | High connectivity | Mid connectivity | Low connectivity | High connectivity | Mid connectivity | Low connectivity | High connectivity | Mid connectivity | Low connectivity | High connectivity | Mid connectivity | Low connectivity |
| Cardiopteridaceae | Citronella paniculata (Mart.) R.A.Howard | 0 | 0 | 0 | 0 | 0 | 0 | 0 | 0 | 0 | 0 | 0 | 1 |
| Anacardiaceae | Astronium graveolens Jacq. | 0 | 0 | 0 | 0 | 0 | 0 | 0 | 0 | 0 | 4 | 0 | 0 |
| Anacardiaceae | Lithraea molleoides (Vell.) Engl. | 0 | 1 | 0 | 0 | 51 | 0 | 0 | 0 | 0 | 0 | 0 | 0 |
| Anacardiaceae | Mangifera indica L | 0 | 0 | 0 | 0 | 1 | 0 | 0 | 0 | 0 | 0 | 0 | 0 |
| Anacardiaceae | Myracrodruon urundeuva Allemão | 0 | 7 | 0 | 0 | 0 | 0 | 0 | 4 | 0 | 0 | 0 | 0 |
| Annonaceae | Annona sylvatica A.St.-Hil. | 0 | 0 | 0 | 5 | 0 | 0 | 0 | 0 | 1 | 0 | 0 | 0 |
| Apocynaceae | Aspidosperma cylindrocarpon Müll.Arg. | 0 | 0 | 0 | 0 | 1 | 0 | 0 | 0 | 0 | 2 | 0 | 0 |
| Apocynaceae | Aspidosperma polyneuron Müll.Arg. | 0 | 0 | 0 | 0 | 0 | 0 | 0 | 0 | 0 | 1 | 2 | 1 |
| Apocynaceae | Aspidosperma ramiflorum Müll.Arg. | 0 | 0 | 0 | 0 | 0 | 0 | 0 | 0 | 0 | 1 | 4 | 1 |
| Arecaceae | Syagrus romanzoffiana (Cham.) Glassman | 0 | 0 | 0 | 0 | 5 | 0 | 0 | 0 | 0 | 0 | 0 | 0 |
| Asteraceae | Moquiniastrum polymorphum (Less.) G. Sancho | 0 | 13 | 11 | 1 | 1 | 0 | 0 | 0 | 0 | 0 | 0 | 0 |
| Bignoniaceae | Handroanthus cf. impetiginosus (Mart. ex DC.) Mattos | 0 | 0 | 0 | 0 | 0 | 0 | 0 | 0 | 0 | 0 | 0 | 1 |
| Bignoniaceae | Handroanthus heptaphyllus (Vell.) Mattos | 0 | 0 | 0 | 0 | 0 | 0 | 0 | 2 | 1 | 0 | 0 | 0 |
| Bignoniaceae | Handroanthus ochraceus (Cham.) Mattos | 0 | 1 | 0 | 0 | 0 | 0 | 0 | 0 | 0 | 0 | 0 | 0 |
| Boraginaceae | Cordia americana (L.) Gottschling & J.S.Mill. | 0 | 1 | 0 | 0 | 0 | 0 | 0 | 0 | 0 | 0 | 0 | 0 |
| Boraginaceae | Cordia ecalyculata Vell. | 0 | 0 | 0 | 0 | 0 | 0 | 0 | 0 | 0 | 1 | 0 | 1 |
| Boraginaceae | Cordia trichotoma (Vell.) Arráb. ex Steud. | 0 | 1 | 0 | 0 | 0 | 0 | 0 | 0 | 0 | 0 | 0 | 0 |
| Cannabaceae | Celtis cf. iguanaea (Jacq.) Sarg. | 0 | 0 | 0 | 0 | 0 | 0 | 2 | 1 | 0 | 0 | 0 | 0 |
| Cannabaceae | Celtis ehrenbergiana (Klotzsch) Liebm. | 0 | 0 | 0 | 0 | 0 | 0 | 0 | 0 | 0 | 0 | 1 | 0 |
| Cannabaceae | Celtis sp. 1 | 0 | 0 | 0 | 0 | 0 | 0 | 0 | 0 | 1 | 0 | 0 | 0 |
| Ebenaceae | Diospyros inconstans Jacq. | 2 | 0 | 0 | 0 | 0 | 0 | 0 | 0 | 0 | 0 | 0 | 0 |
| Elaeocarpaceae | Sloanea lasiocoma K.Schum. | 0 | 0 | 0 | 0 | 0 | 0 | 0 | 0 | 0 | 0 | 0 | 2 |
| Euphorbiaceae | Actinostemon concepcionis (Chodat & Hassl.) Hochr. | 0 | 0 | 0 | 0 | 0 | 0 | 0 | 0 | 0 | 3 | 1 | 1 |
| Euphorbiaceae | Alchornea glandulosa Poepp. & Endl. | 1 | 0 | 2 | 2 | 0 | 6 | 0 | 0 | 0 | 0 | 0 | 0 |
| Euphorbiaceae | Croton floribundus Spreng. | 0 | 0 | 1 | 3 | 0 | 0 | 12 | 0 | 8 | 0 | 0 | 0 |
| Euphorbiaceae | Croton urucurana Baill. | 18 | 0 | 0 | 0 | 1 | 0 | 0 | 0 | 0 | 0 | 0 | 0 |
| Euphorbiaceae | Gymnanthes klotzschiana Müll.Arg. | 0 | 0 | 0 | 0 | 0 | 0 | 0 | 0 | 0 | 12 | 0 | 0 |
| Euphorbiaceae | Gymnanthes nervosa Müll.Arg. | 0 | 0 | 0 | 0 | 0 | 0 | 0 | 0 | 0 | 3 | 0 | 0 |
| Euphorbiaceae | Sebastiania brasiliensis Spreng. | 0 | 0 | 4 | 1 | 0 | 0 | 0 | 0 | 0 | 0 | 0 | 0 |
| Fabaceae | Albizia niopoides (Spruce ex Benth.) Burkart | 0 | 0 | 0 | 0 | 0 | 0 | 0 | 0 | 5 | 0 | 0 | 0 |
| Fabaceae | Andira fraxinifolia Benth. | 0 | 0 | 9 | 0 | 0 | 0 | 0 | 0 | 0 | 0 | 0 | 0 |
| Fabaceae | Bauhinia longifolia (Bong.) Steud. | 0 | 0 | 0 | 0 | 0 | 0 | 0 | 0 | 7 | 0 | 0 | 0 |
| Fabaceae | Centrolobium tomentosum Guillem. ex Benth. | 0 | 0 | 0 | 1 | 3 | 0 | 2 | 0 | 0 | 0 | 1 | 0 |
| Fabaceae | Copaifera langsdorffii Desf. | 0 | 0 | 0 | 1 | 0 | 0 | 0 | 0 | 4 | 2 | 0 | 0 |
| Fabaceae | Dahlstedtia muehlbergiana (Hassl.) M.J.Silva & A.M.G. Azevedo | 0 | 0 | 0 | 0 | 0 | 0 | 0 | 0 | 44 | 0 | 0 | 0 |
| Fabaceae | Enterolobium contortisiliquum (Vell.) Morong | 2 | 1 | 2 | 0 | 2 | 0 | 0 | 2 | 0 | 0 | 0 | 0 |
| Fabaceae | Holocalyx balansae Micheli | 0 | 0 | 0 | 0 | 0 | 0 | 0 | 0 | 0 | 0 | 3 | 1 |
| Fabaceae | Hymenaea courbaril L. | 0 | 0 | 0 | 0 | 0 | 0 | 0 | 0 | 0 | 1 | 0 | 0 |
| Fabaceae | Leucaena leucocephala (Lam.) de Wit | 7 | 0 | 0 | 0 | 0 | 0 | 0 | 0 | 0 | 0 | 0 | 0 |
| Fabaceae | Leucochloron incuriale (Vell.) Barneby & J.W.Grimes | 0 | 0 | 1 | 0 | 0 | 0 | 0 | 0 | 0 | 0 | 0 | 0 |
| Fabaceae | Lonchocarpus cultratus (Vell.) A.M.G.Azevedo & H.C.Lima | 1 | 3 | 0 | 0 | 0 | 2 | 0 | 0 | 0 | 0 | 0 | 0 |
| Fabaceae | Machaerium brasiliense Vogel | 0 | 2 | 0 | 0 | 0 | 0 | 0 | 11 | 7 | 0 | 0 | 0 |
| Fabaceae | Machaerium hirtum (Vell.) Stellfeld | 1 | 1 | 1 | 0 | 2 | 3 | 0 | 0 | 0 | 0 | 0 | 0 |
| Fabaceae | Machaerium nyctitans (Vell.) Benth. | 0 | 20 | 1 | 20 | 1 | 0 | 2 | 0 | 0 | 0 | 0 | 1 |
| Fabaceae | Machaerium stipitatum Vogel | 0 | 0 | 0 | 0 | 0 | 1 | 0 | 9 | 5 | 0 | 0 | 1 |
| Fabaceae | Machaerium villosum Vogel | 0 | 4 | 0 | 2 | 0 | 1 | 0 | 0 | 2 | 0 | 0 | 0 |
| Fabaceae | Myroxylon peruiferum L.f. | 0 | 0 | 0 | 0 | 0 | 0 | 2 | 1 | 0 | 0 | 0 | 0 |
| Fabaceae | Peltophorum dubium (Spreng.) Taub. | 0 | 0 | 0 | 0 | 0 | 0 | 0 | 2 | 0 | 0 | 0 | 0 |
| Fabaceae | Piptadenia gonoacantha (Mart.) J.F.Macbr. | 0 | 0 | 0 | 0 | 0 | 70 | 0 | 0 | 0 | 0 | 0 | 0 |
| Fabaceae | Platypodium elegans Vogel | 0 | 10 | 4 | 0 | 2 | 0 | 12 | 0 | 1 | 0 | 0 | 0 |
| Fabaceae | Senegalia polyphylla (DC.) Britton & Rose | 0 | 0 | 0 | 0 | 0 | 0 | 0 | 0 | 0 | 1 | 0 | 0 |
| Fabaceae | Senna multijuga (Rich.) H.S.Irwin & Barneby | 0 | 0 | 0 | 0 | 0 | 0 | 0 | 1 | 0 | 0 | 0 | 0 |
| Fabaceae | Sesbania virgata (Cav.) Pers. | 0 | 0 | 0 | 0 | 0 | 0 | 0 | 0 | 0 | 0 | 0 | 1 |
| Lauraceae | Cryptocarya cf. saligna Mez | 0 | 0 | 0 | 0 | 0 | 0 | 0 | 0 | 0 | 1 | 0 | 0 |
| Lauraceae | Lauraceae spp. | 0 | 0 | 0 | 0 | 0 | 0 | 1 | 0 | 0 | 0 | 0 | 0 |
| Lauraceae | Nectandra lanceolata Nees | 0 | 0 | 0 | 0 | 0 | 0 | 0 | 0 | 2 | 0 | 0 | 0 |
| Lauraceae | Ocotea indecora (Schott) Mez | 0 | 0 | 0 | 0 | 0 | 0 | 0 | 0 | 0 | 6 | 0 | 0 |
| Lauraceae | Ocotea odorifera (Vell.) Rohwer | 0 | 0 | 0 | 0 | 0 | 0 | 0 | 0 | 0 | 0 | 0 | 1 |
| Lauraceae | Ocotea puberula  (Rich.) Nees | 0 | 0 | 0 | 0 | 0 | 1 | 0 | 0 | 6 | 0 | 0 | 0 |
| Lecythidaceae | Cariniana estrellensis (Raddi) Kuntze | 1 | 1 | 0 | 1 | 0 | 0 | 0 | 0 | 2 | 2 | 0 | 3 |
| Lecythidaceae | Cariniana legalis (Mart.) Kuntze | 0 | 0 | 0 | 0 | 0 | 0 | 0 | 0 | 0 | 1 | 1 | 1 |
| Lythraceae | Lafoensia pacari A.St.-Hil. | 0 | 0 | 0 | 1 | 0 | 0 | 0 | 0 | 0 | 0 | 0 | 0 |
| Malvaceae | Bastardiopsis densiflora (Hook. & Arn.) Hassl. | 0 | 0 | 0 | 0 | 0 | 0 | 1 | 0 | 0 | 0 | 0 | 0 |
| Malvaceae | Ceiba speciosa (A.St.-Hil.) Ravenna | 0 | 0 | 0 | 0 | 0 | 0 | 0 | 1 | 0 | 1 | 0 | 0 |
| Malvaceae | Guazuma ulmifolia Lam. | 11 | 9 | 0 | 1 | 6 | 0 | 3 | 0 | 1 | 0 | 0 | 0 |
| Malvaceae | Luehea candicans Mart. & Zucc. | 0 | 49 | 0 | 2 | 17 | 0 | 11 | 2 | 1 | 0 | 0 | 0 |
| Malvaceae | Luehea grandiflora Mart. & Zucc. | 0 | 0 | 0 | 0 | 1 | 0 | 0 | 0 | 0 | 0 | 0 | 0 |
| Malvaceae | Luehea paniculata Mart. & Zucc. | 1 | 0 | 0 | 1 | 0 | 0 | 0 | 0 | 0 | 0 | 0 | 0 |
| Melastomataceae | Miconia albicans (Sw.) Triana | 0 | 0 | 4 | 0 | 0 | 0 | 0 | 0 | 0 | 0 | 0 | 0 |
| Meliaceae | Cabralea canjerana (Vell.) Mart. | 0 | 0 | 0 | 0 | 0 | 0 | 0 | 0 | 1 | 0 | 0 | 1 |
| Meliaceae | Cedrela fissilis Vell. | 0 | 0 | 0 | 0 | 1 | 1 | 1 | 0 | 0 | 0 | 0 | 0 |
| Meliaceae | Guarea guidonia (L.) Sleumer | 0 | 0 | 0 | 2 | 0 | 0 | 0 | 0 | 0 | 0 | 0 | 0 |
| Meliaceae | Guarea macrophylla Vahl | 2 | 0 | 0 | 0 | 0 | 3 | 1 | 0 | 8 | 0 | 0 | 0 |
| Meliaceae | Trichilia casaretti C.DC. | 0 | 0 | 0 | 0 | 0 | 0 | 0 | 0 | 0 | 0 | 30 | 0 |
| Meliaceae | Trichilia catigua A.Juss. | 0 | 0 | 0 | 0 | 0 | 0 | 1 | 0 | 0 | 0 | 2 | 0 |
| Meliaceae | Trichilia cf. clausseni C.DC. | 0 | 0 | 0 | 0 | 0 | 0 | 0 | 0 | 0 | 0 | 1 | 0 |
| Meliaceae | Trichilia clausseni C.DC. | 0 | 0 | 0 | 0 | 2 | 0 | 20 | 14 | 0 | 0 | 0 | 1 |
| Meliaceae | Trichilia pallida Sw. | 0 | 0 | 0 | 1 | 0 | 12 | 2 | 0 | 4 | 1 | 0 | 0 |
| Monimiaceae | Mollinedia widgrenii A.DC. | 0 | 0 | 0 | 0 | 0 | 0 | 1 | 0 | 1 | 0 | 0 | 0 |
| Moraceae | Ficus guaranitica Chodat | 0 | 0 | 0 | 0 | 0 | 0 | 0 | 0 | 0 | 0 | 1 | 0 |
| Moraceae | Maclura tinctoria (L.) D.Don ex Steud. | 0 | 1 | 0 | 0 | 0 | 0 | 0 | 0 | 0 | 0 | 0 | 1 |
| morta | morta | 0 | 3 | 0 | 0 | 0 | 0 | 0 | 0 | 0 | 0 | 0 | 0 |
| Myrtaceae | Calyptranthes sp. 1 | 0 | 0 | 0 | 0 | 0 | 0 | 0 | 0 | 0 | 1 | 0 | 0 |
| Myrtaceae | Calyptranthes spp. | 0 | 0 | 0 | 0 | 0 | 0 | 0 | 0 | 0 | 0 | 0 | 1 |
| Myrtaceae | Campomanesia xanthocarpa (Mart.) O.Berg | 0 | 0 | 0 | 6 | 0 | 0 | 0 | 0 | 0 | 0 | 0 | 0 |
| Myrtaceae | Eucalyptus spp. | 0 | 0 | 4 | 0 | 0 | 2 | 0 | 0 | 0 | 0 | 0 | 0 |
| Myrtaceae | Eugenia dodonaeifolia Cambess. | 0 | 0 | 0 | 1 | 0 | 0 | 0 | 0 | 0 | 0 | 0 | 1 |
| Myrtaceae | Eugenia florida DC. | 0 | 0 | 10 | 1 | 0 | 0 | 3 | 0 | 0 | 0 | 0 | 1 |
| Myrtaceae | Eugenia ligustrina (Sw.) Willd. | 0 | 0 | 0 | 0 | 0 | 0 | 0 | 0 | 0 | 4 | 0 | 0 |
| Myrtaceae | Eugenia sp. 1 | 0 | 0 | 0 | 0 | 0 | 0 | 0 | 0 | 0 | 1 | 0 | 0 |
| Myrtaceae | Eugenia sp. 3 | 0 | 0 | 0 | 0 | 0 | 0 | 0 | 0 | 0 | 1 | 0 | 0 |
| Myrtaceae | Eugenia spp. | 0 | 0 | 0 | 0 | 0 | 0 | 0 | 0 | 0 | 0 | 0 | 3 |
| Myrtaceae | Myrcia sp. 4 | 0 | 0 | 0 | 0 | 0 | 0 | 0 | 0 | 0 | 1 | 0 | 0 |
| Myrtaceae | Myrcia splendens (Sw.) DC. | 0 | 0 | 0 | 0 | 0 | 0 | 0 | 0 | 0 | 1 | 0 | 0 |
| Myrtaceae | Myrcia tomentosa (Aubl.) DC. | 0 | 0 | 1 | 0 | 0 | 0 | 0 | 0 | 0 | 0 | 0 | 0 |
| Myrtaceae | Myrciaria floribunda (H.West ex Willd.) O.Berg | 0 | 0 | 0 | 1 | 0 | 0 | 0 | 0 | 0 | 0 | 0 | 0 |
| Myrtaceae | Myrtaceae 1 | 0 | 0 | 0 | 0 | 0 | 0 | 0 | 0 | 0 | 1 | 0 | 0 |
| Myrtaceae | Plinia peruviana (Poir.) Govaerts | 0 | 0 | 0 | 0 | 0 | 1 | 0 | 0 | 0 | 2 | 0 | 0 |
| Myrtaceae | Psidium guajava L. | 38 | 13 | 4 | 0 | 17 | 0 | 0 | 1 | 0 | 0 | 0 | 0 |
| Myrtaceae | Psidium sartorianum (O.Berg) Nied. | 0 | 0 | 0 | 8 | 0 | 0 | 0 | 0 | 0 | 0 | 0 | 0 |
| Nyctaginaceae | Guapira hirsuta (Choisy) Lundell | 0 | 0 | 0 | 0 | 0 | 0 | 0 | 0 | 1 | 0 | 0 | 0 |
| Nyctaginaceae | Guapira opposita (Vell.) Reitz | 0 | 0 | 0 | 0 | 0 | 0 | 0 | 1 | 0 | 0 | 0 | 1 |
| Peraceae | Pera glabrata (Schott) Poepp. ex Baill. | 0 | 0 | 1 | 0 | 0 | 0 | 0 | 0 | 0 | 0 | 0 | 0 |
| Phyllanthaceae | Savia dictyocarpa Müll.Arg. | 0 | 0 | 0 | 0 | 0 | 0 | 0 | 0 | 0 | 17 | 4 | 0 |
| Proteaceae | Roupala montana Aubl. | 0 | 0 | 0 | 0 | 0 | 0 | 0 | 0 | 1 | 3 | 0 | 0 |
| Rhamnaceae | Rhamnidium elaeocarpum Reissek | 0 | 0 | 0 | 0 | 0 | 0 | 2 | 3 | 1 | 0 | 0 | 0 |
| Rubiaceae | Coutarea hexandra (Jacq.) K.Schum. | 0 | 0 | 0 | 1 | 0 | 0 | 0 | 0 | 0 | 0 | 0 | 0 |
| Rubiaceae | Rudgea jasminoides (Cham.) Müll.Arg. | 0 | 0 | 0 | 0 | 0 | 0 | 0 | 0 | 0 | 6 | 0 | 0 |
| Rutaceae | Conchocarpus pentandrus (A. St.-Hil.) Kallunki & Pirani | 0 | 0 | 0 | 0 | 0 | 0 | 0 | 0 | 0 | 2 | 15 | 0 |
| Rutaceae | Esenbeckia febrifuga (A.St.-Hil.) A. Juss. ex Mart. | 0 | 1 | 0 | 1 | 0 | 1 | 0 | 0 | 0 | 0 | 0 | 0 |
| Rutaceae | Esenbeckia leiocarpa Engl. | 0 | 0 | 0 | 0 | 0 | 0 | 0 | 0 | 0 | 0 | 9 | 0 |
| Rutaceae | Galipea jasminiflora (A.St.-Hil.) Engl. | 0 | 0 | 0 | 0 | 0 | 0 | 0 | 0 | 0 | 7 | 0 | 37 |
| Rutaceae | Metrodorea nigra A.St.-Hil. | 0 | 0 | 0 | 0 | 0 | 0 | 0 | 0 | 0 | 35 | 0 | 1 |
| Rutaceae | Zanthoxylum caribaeum Lam. | 0 | 0 | 0 | 0 | 0 | 0 | 0 | 0 | 0 | 0 | 0 | 6 |
| Rutaceae | Zanthoxylum monogynum A.St.-Hil. | 0 | 0 | 10 | 0 | 0 | 0 | 0 | 0 | 0 | 0 | 0 | 0 |
| Rutaceae | Zanthoxylum rhoifolium Lam. | 0 | 0 | 1 | 3 | 0 | 1 | 7 | 1 | 0 | 0 | 0 | 0 |
| Rutaceae | Zanthoxylum riedelianum Engl. | 0 | 2 | 0 | 0 | 0 | 0 | 0 | 0 | 1 | 0 | 0 | 0 |
| Salicaceae | Casearia decandra Jacq. | 0 | 0 | 0 | 1 | 0 | 0 | 1 | 0 | 0 | 0 | 0 | 0 |
| Salicaceae | Casearia gossypiosperma Briq. | 0 | 0 | 0 | 12 | 0 | 0 | 3 | 0 | 0 | 1 | 1 | 0 |
| Salicaceae | Casearia sylvestris Sw. | 0 | 15 | 2 | 11 | 1 | 3 | 4 | 1 | 7 | 1 | 0 | 0 |
| Salicaceae | Prockia crucis P.Browne ex L. | 0 | 0 | 0 | 0 | 0 | 0 | 0 | 1 | 0 | 0 | 0 | 0 |
| Sapindaceae | Allophylus edulis (A.St.-Hil. et al.) Hieron. ex Niederl. | 0 | 0 | 0 | 0 | 5 | 0 | 2 | 1 | 1 | 0 | 0 | 0 |
| Sapindaceae | Allophylus racemosus Sw. | 0 | 0 | 0 | 0 | 0 | 1 | 0 | 2 | 0 | 0 | 0 | 0 |
| Sapindaceae | Cupania vernalis Cambess. | 0 | 4 | 0 | 0 | 0 | 0 | 0 | 0 | 0 | 0 | 0 | 0 |
| Sapotaceae | Chrysophyllum gonocarpum (Mart. & Eichler ex Miq.) Engl. | 0 | 0 | 0 | 0 | 0 | 1 | 0 | 0 | 0 | 2 | 0 | 2 |
| Sapotaceae | Chrysophyllum marginatum (Hook. & Arn.) Radlk. | 0 | 0 | 0 | 1 | 0 | 0 | 2 | 0 | 0 | 0 | 0 | 0 |
| Sapotaceae | Pouteria sp. 1 | 0 | 0 | 0 | 0 | 0 | 0 | 0 | 0 | 0 | 0 | 0 | 3 |
| Siparunaceae | Siparuna guianensis Aubl. | 0 | 0 | 0 | 0 | 0 | 1 | 0 | 0 | 0 | 0 | 0 | 0 |
| Solanaceae | Acnistus arborescens (L.) Schltdl. | 1 | 0 | 0 | 0 | 0 | 0 | 0 | 0 | 0 | 0 | 0 | 0 |
| Solanaceae | Cestrum intermedium Sendtn. | 0 | 0 | 0 | 0 | 0 | 1 | 0 | 0 | 0 | 0 | 0 | 0 |
| Unclassified | Unclassified | 0 | 0 | 1 | 1 | 0 | 0 | 2 | 3 | 6 | 2 | 0 | 1 |
| Urticaceae | Cecropia pachystachya Trécul | 1 | 0 | 1 | 0 | 0 | 0 | 0 | 0 | 0 | 0 | 0 | 0 |
| Urticaceae | Urera caracasana (Jacq.) Griseb. | 0 | 0 | 0 | 0 | 0 | 1 | 0 | 0 | 0 | 0 | 0 | 0 |
| Verbenaceae | Aloysia virgata (Ruiz & Pav.) Juss. | 2 | 2 | 0 | 0 | 3 | 0 | 4 | 23 | 0 | 0 | 0 | 0 |
| Verbenaceae | Citharexylum myrianthum Cham. | 1 | 0 | 0 | 0 | 0 | 0 | 1 | 0 | 0 | 0 | 0 | 0 |
| Vochysiaceae | Vochysia tucanorum Mart. | 0 | 2 | 0 | 0 | 0 | 0 | 0 | 0 | 0 | 0 | 0 | 0 |

**References**

Barbi, F., Martinovi, T., Holl, S. A., Lepinay, C., Lenka, M., Baldrian, P., Kohout, P., & Thoen, E. (2025). *Disentangling drivers behind fungal diversity gradients along altitude and latitude*. https://doi.org/10.1111/nph.70012

Beidler, K. V., & Pritchard, S. G. (2017). Maintaining connectivity: understanding the role of root order and mycelial networks in fine root decomposition of woody plants. In *Plant and Soil* (Vol. 420, Issues 1–2, pp. 19–36). Springer International Publishing. https://doi.org/10.1007/s11104-017-3393-8

Bieluczyk, W., Duarte, M. P., Martins, G. L., Mandro, J. A., Camargo, P. B. de, Noronha, N. C., Piccolo, M. de C., & Tsai, S. M. (2025). Slash-and-burn agriculture disrupts the carbon storage potential and ecosystem multifunctionality of Amazon’s secondary forests. *Agriculture, Ecosystems and Environment*, *381*. https://doi.org/10.1016/j.agee.2024.109413

César, R. G., Moreno, V. de S., Coletta, G. D., Schweizer, D., Chazdon, R. L., Barlow, J., Ferraz, S. F. B., Crouzeilles, R., & Brancalion, P. H. S. (2021). It is not just about time: Agricultural practices and surrounding forest cover affect secondary forest recovery in agricultural landscapes. *Biotropica*, *53*(2), 496–508. https://doi.org/10.1111/btp.12893

Garcia, L. C., Hobbs, R. J., Ribeiro, D. B., Tamashiro, J. Y., Santos, F. A. M., & Rodrigues, R. R. (2016). Restoration over time: is it possible to restore trees and non-trees in high-diversity forests? *Applied Vegetation Science*, *19*(4), 655–666. https://doi.org/10.1111/avsc.12264

Getaneh, S., Honnay, O., Desie, E., Helsen, K., Couck, L., Shibru, S., & Muys, B. (2022). Impact of tree litter identity, litter diversity and habitat quality on litter decomposition rates in tropical moist evergreen forest. *Forest Ecosystems*, *9*. https://doi.org/10.1016/j.fecs.2022.100023

Hannula, S. E., Morriën, E., De Hollander, M., Van Der Putten, W. H., Van Veen, J. A., & De Boer, W. (2017). Shifts in rhizosphere fungal community during secondary succession following abandonment from agriculture. *ISME Journal*, *11*(10), 2294–2304. https://doi.org/10.1038/ismej.2017.90

Horodecki, P., Nowiński, M., & Jagodziński, A. M. (2019). Advantages of mixed tree stands in restoration of upper soil layers on postmining sites: A five-year leaf litter decomposition experiment. *Land Degradation and Development*, *30*(1), 3–13. https://doi.org/10.1002/ldr.3194

Lanuza, O., Casanoves, F., Delgado, D., & Van den Meersche, K. (2019). Leaf litter stoichiometry affects decomposition rates and nutrient dynamics in tropical forests under restoration in Costa Rica. *Restoration Ecology*, *27*(3), 549–558. https://doi.org/10.1111/rec.12893

Odriozola, I., Martinović, T., Mašínová, T., Bahnmann, B. D., Machac, A., Sedlák, P., Tomšovský, M., & Baldrian, P. (2023). The spatial patterns of community composition, their environmental drivers and their spatial scale dependence vary markedly between fungal ecological guilds. *Global Ecology and Biogeography*. https://doi.org/10.1111/geb.13772

Rother, D. C., Vidal, C. Y., Fagundes, I. C., Metran da Silva, M., Gandolfi, S., Rodrigues, R. R., Nave, A. G., Viani, R. A. G., & Brancalion, P. H. S. (2018). How Legal-Oriented Restoration Programs Enhance Landscape Connectivity? Insights From the Brazilian Atlantic Forest. *Tropical Conservation Science*, *11*. https://doi.org/10.1177/1940082918785076

Schnabel, F., Guillemot, J., Barry, K. E., Brunn, M., Cesarz, S., Eisenhauer, N., Gebauer, T., Guerrero-Ramirez, N. R., Handa, I. T., Madsen, C., Mancilla, Lady, Monteza, J., Moore, T., Oelmann, Y., Scherer-Lorenzen, M., Schwendenmann, L., Wagner, A., Wirth, C., & Potvin, C. (2025). Tree Diversity Increases Carbon Stocks and Fluxes Above—But Not Belowground in a Tropical Forest Experiment. *Global Change Biology*, *31*(2). https://doi.org/10.1111/gcb.70089

Urbanová, M., Šnajdr, J., & Baldrian, P. (2015). Composition of fungal and bacterial communities in forest litter and soil is largely determined by dominant trees. *Soil Biology and Biochemistry*, *84*, 53–64. https://doi.org/10.1016/j.soilbio.2015.02.011

Yang, W., Diao, L., Wang, Y., Yang, X., Zhang, H., Wang, J., Luo, Y., An, S., & Cheng, X. (2022). Responses of soil fungal communities and functional guilds to ~160 years of natural revegetation in the Loess Plateau of China. *Frontiers in Microbiology*, *13*. https://doi.org/10.3389/fmicb.2022.967565

Zhang, X., Wang, L., Zhou, W., Hu, W., Hu, J., & Hu, M. (2022). Changes in litter traits induced by vegetation restoration accelerate litter decomposition in Robinia pseudoacacia plantations. *Land Degradation and Development*, *33*(1), 179–192. https://doi.org/10.1002/ldr.4136
